# Phenotypic Differences in Virulence and Immune Response in Closely Related Clinical Isolates of Influenza A 2009 H1N1 Pandemic Viruses in Mice

**DOI:** 10.1371/journal.pone.0056602

**Published:** 2013-02-18

**Authors:** Jeremy V. Camp, Yong-Kyu Chu, Dong-Hoon Chung, Ryan C. McAllister, Robert S. Adcock, Rachael L. Gerlach, Timothy L. Wiemken, Paula Peyrani, Julio A. Ramirez, James T. Summersgill, Colleen B. Jonsson

**Affiliations:** 1 Department of Microbiology and Immunology, University of Louisville, Kentucky, United States of America; 2 Center for Predictive Medicine for Biodefense and Emerging Infectious Diseases, University of Louisville, Kentucky, United States of America; 3 Division of Infectious Diseases, Department of Medicine, University of Louisville, Louisville, Kentucky, United States of America; University of Ottawa, Canada

## Abstract

To capture the possible genotypic and phenotypic differences of the 2009 influenza A virus H1N1 pandemic (H1N1pdm) strains circulating in adult hospitalized patients, we isolated and sequenced nine H1N1pdm viruses from patients hospitalized during 2009–2010 with severe influenza pneumonia in Kentucky. Each viral isolate was characterized in mice along with two additional H1N1 pandemic strains and one seasonal strain to assess replication and virulence. All isolates showed similar levels of replication in nasal turbinates and lung, but varied in their ability to cause morbidity. Further differences were identified in cytokine and chemokine responses. IL-6 and KC were expressed early in mice infected with strains associated with higher virulence. Strains that showed lower pathogenicity in mice had greater IFNγ, MIG, and IL-10 responses. A principal component analysis (PCA) of the cytokine and chemokine profiles revealed 4 immune response phenotypes that correlated with the severity of disease. A/KY/180/10, which showed the greatest virulence with a rapid onset of disease progression, was compared in additional studies with A/KY/136/09, which showed low virulence in mice. Analyses comparing a low (KY/136) versus a high (KY/180) virulent isolate showed a significant difference in the kinetics of infection within the lower respiratory tract and immune responses. Notably by 4 DPI, virus titers within the lung, bronchoalveolar lavage fluid (BALf), and cells within the BAL (BALc) revealed that the KY/136 replicated in BALc, while KY/180 replication persisted in lungs and BALc. In summary, our studies suggest four phenotypic groups based on immune responses that result in different virulence outcomes in H1N1pdm isolates with a high degree of genetic similarity. *In vitro* studies with two of these isolates suggested that the more virulent isolate, KY/180, replicates productively in macrophages and this may be a key determinant in tipping the response toward a more severe disease progression.

## Introduction

The 2009 pandemic H1N1 influenza virus (H1N1pdm) arose through reassortment of two preexisting swine influenza viruses, a Eurasian avian-like virus and a North American triple reassortant virus [1]–[3]. The risk factors associated with human cases of H1N1pdm mirrored those of seasonal influenza [4]. As observed with seasonal influenza, the most common underlying chronic conditions among hospitalized patients were respiratory disease, asthma, cardiac disease, and diabetes [4]–[9]. However, in contrast to seasonal influenza, a greater proportion of severe and fatal cases had a pre-existing chronic illness. A second notable difference was the age distribution of hospitalized and severe cases. Children less than 17 years old had the greatest rates of hospitalization per capita and adults over 64 had the greatest rates of mortality per capita. Retrospective clinical studies focused on surveillance of H1N1pdm sequences present in ICU admissions suggest that pre-existing medical conditions may be a more important factor in severity rather than particular viral variants [10]. However, this does not explain why a greater proportion of persons with pre-existing medical conditions had more severe disease than typically observed for seasonal influenza. Further, previously unreported comorbidities such as morbid obesity have been widely suggested for H1N1pdm for increased risk for admission to the ICU and death [11]. At this time, it is difficult to rule out the contribution of viral variants to the resulting illness observed with the various comorbidities. Nonetheless, the course of illness and the progression to more severe disease are most likely due to the combined interplay of the individual’s health, the intrinsic phenotype of the infecting viral variant and the treatment regime.

In contrast to seasonal influenza viruses, the H1N1pdm viruses replicate well and show greater pathogenicity with viral antigen in the bronchiolar epithelia and the alveolus by day 3 post-infection (DPI) [2], [3], [12]. In the BALB/c mouse, A/California/04/09 and other H1N1pdm viruses show lethality, but only at the highest dose of 10^6.5^ plaque forming units (PFU) [2], [12]. Studies of infection of BALB/c with several different 2009 H1N1pdm virus isolates show high virus titers in nasal turbinates (NT) and lung tissues by 3 DPI. Virus titers show a slight decrease by 6 DPI in lung and NT although the decrease varies among strains, too. Proinflammatory cytokines and chemokines were elevated for most mice in whole lung specimens at 3DPI for KC, IL-6, IL-12(p40), G-CSF, M-CSF, MCP-1 MIG, MIP-1β, and LIF. By 6 DPI, IL-10 was present, albeit low, whereas some of the cytokines were reduced (e.g., MIP-2, IL-6), but most remained elevated [2], [12]. The levels of cytokines and chemokines in humans or mice do not suggest hypercytokinemia common to H5N1 and 1918 viruses [13]–[15].

The overall genetic distance among H1N1pdm isolates remained low with 7 distinct clades in the first wave of the pandemic, while in the second wave a single viral lineage dominated [16]. Molecular surveillance, while important, will not confirm potential phenotypic differences *in vivo* resulting from amino acid changes associated with viral variants in newly emerging strains or the potential for mixed infections and the genetic diversity of the intrahost viral populations [17]. Animal studies can complement molecular surveillance in monitoring the potential pathogenicity and immunogenicity of circulating strains. Further, such studies have potential to reveal the efficacy of treatment regimes. Herein we report the isolation, sequence, and characterization of nine H1N1pdm influenza A viruses from adult patients hospitalized in Kentucky during the second pandemic wave, September 2009 and April 2010. Four of the nine patients died and all of the patients for whom data was available had an underlying chronic condition. This group of clinical isolates, with high genetic similarity, was characterized for virus load, virulence, and host immune response in mice. Immune responses in the lungs suggest four distinct immunological phenotypes that correlate with the observed mortality in mice. This study underscores the potential variability in the virulence of 2009 H1N1 influenza A strains circulating in Kentucky during the pandemic. These data suggest the hypothesis that the high severity of disease seen in certain hospitalized patients may be related to infection with H1N1pdm viral variants that, due to cell tropism and replication levels, may exacerbate certain types of disease associated with comorbidity.

## Results

### Isolation and Sequence Analyses of H1N1pdm Isolates from Hospitalized Patients

H1N1pdm strains were isolated from nasopharyngeal swab samples obtained from nine de-identified patients enrolled in the Severe Influenza Pneumonia Surveillance (SIPS) project, a clinical study of hospitalized patients with severe community-acquired pneumonia in Kentucky from December 2008– December 2011 (Table 1). Hospitals included in the SIPS project included two rural areas of Kentucky, one in the east and one in the midwest, and one in an urban area (Louisville). The midwest is active in agriculture and one of the highest livestock producers of hogs and pigs. The age of the patients ranged from 31 to 58 and included 5 females and 4 males. Many of those patients had underlying comorbidities commonly associated with severe influenza disease such as obesity, diabetes and chronic obstructive pulmonary disorder (COPD) (Table S1). Four of the nine patients died.

**Table 1:** General Patient Data for Nasal Swabs Used for Virus Isolation.

Sample ID/Locality Code*	Patient Age Sex	Hospital Admission Date	Nasal Swab Date	LOS Ψ (Days)	Comorbidity	Mortality
80/3	31/F	9/30/2009	9/30/2009	10	COPD, MRSA	Died
96/2	51/M	10/24/2009	10/26/2009	4	BMI = 123.6	Survived
99/3	35/M	10/28/2009	10/29/2009	3	ND	Survived
104/2	58/M	10/30/2009	11/4/2009	2	ND	Died
108/6	57/F	11/2/2009	11/3/2009	19	ND	Survived
110/6	54/F	11/3/2009	11/4/2009	3	COPD, diabetes, renal disease, BMI = 79.7	Survived
136/3	46/M	12/10/2009	12/11/2009	8	Diabetes, MRSA	Survived
180/2	53/M	3/24/2010	4/1/2010	19	COPD, renal disease	Died
190/2	55/F	4/10/2010	4/15/2010	12	ND	Died

* locality, 2 = western Kentucky (KY); 3 = eastern KY; 6 = Louisville metro;

Ψ LOS, Length of stay in hospital; MRSA, methicillin-Resistant *Staphylococcus aureus*; COPD, chronic obstructive pulmonary disease; BMI- body mass index; ND, none determined.

Viruses were isolated by passage through both Madin-Darby canine kidney (MDCK) cells and eggs and virus isolates were designated according to a tracking number that contains no patient identifier information. Herein, isolates will be referred to by the tracking number and virus isolation method (*e.g.,* A/Kentucky/180/2010 egg-passaged isolate will be referred to as “KY/180E”). Viruses were amplified and the virus titers were determined by 50% tissue culture infectious dose (TCID_50_), plaque forming units (PFU) and hemagglutination assays in MDCK cells (data not shown). Viral titers measured in TCID_50_ and PFU assays were similar for MDCK-adapted viruses and egg-adapted isolates (e.g., median titer ∼10^7^). Some viruses (A/KY/96/09, A/KY/99/09, A/KY/108/09, A/KY/180/10) showed a 10–100 fold higher titers in MDCK cell culture than in egg, but some (A/KY/80/09, KY/110/09, KY/136/09) did not. Egg-adapted viruses that were not recovered included A/KY/104/09 and A/KY/190/10. Hemagglutination titers were generally lower from egg-adapted viruses and showed variability among isolates (data not shown).

Universal and gene-specific primers were designed and used to amplify and sequence full-length cDNAs of all viral segments from each isolate. The amino acid sequences encoded by each gene were deduced and compared. All of the isolates have stop codons in PB1-F2 at positions 12, 58, and 88 (data not shown) [18]. Amino acids that showed polymorphisms are listed for each viral protein for the majority of genes (Table S2). The most amino acid changes were observed in HA1 and acidic polymerase (PA) with 18 and 9 mutations collectively across the 7–8 the KY isolates (not all isolates were completely sequenced). Neuraminidase and the two basic polymerase subunits (NA, PB1 and PB2) each had 4–5 mutations while the nonstructural protein 1 (NS1) and HA2 showed 3 mutations collectively. In contrast, matrix (M1), nucleoprotein (NP) had only 1 mutation and there were none in M2, NS2 or PA-X. Of the mutations only a few have been previously studied functionally to our knowledge. For example, in the HA1/HA2 of KY/180E we noted changes in D222G, S83P, S183P, and E374K. E374K was identified as a vaccine escape mutation [19]. The S183P and the Q293H mutations in HA1 (found in KY/180E and KY/96E, respectively) have been reported by others [20]–[23], and have been associated with alterations in receptor binding and increases in disease severity in humans [24], [25].

### Differential Progression of Virus Replication and Disease in Mice Infected with H1N1pdm Strains

To make an initial assessment of the levels of replication and virulence potential of each of the clinical H1N1pdm isolates, groups of six DBA2 mice were intranasally-infected and monitored daily for clinical signs and body weight. In addition to the nine clinical isolates, we included two pandemic strains (A/CA/07/09 and A/NY/18/09) and a seasonal H1N1 strain (A/BN/59/07). On days three and six, three mice each were euthanized and analyzed for viral load and antibody titers (Table 2). All mice infected with influenza strains showed weight loss (Table 2). Mice infected with KY/96M, KY/80M, KY/180E, KY/80E, and NY/18E showed the greatest weight loss in this short study (Table 2). Mice infected with the seasonal influenza strain BN/59E showed the least weight loss (about 9%). All other mice infected with H1N1pdm influenza isolates (KY/136M, KY/99M, KY/136E, KY/110E, CA/07E, KY/108M, KY/110M, KY/104M, and KY/108E) showed weight loss averages ranging from 13% to 26%, respectively.

**Table 2:** Summary of viral titers, lethality of H1N1pdm isolates in DBA2 mice.

Inoculum	DPI	Virus titer (Lung)	Lethality
Mock	3	<1.5 *	0.0
	6	<1.5 *	0.0
KY/80M	3	7.2±0.5	0.0
	6	6.4±0.9	33.3
KY/80E	3	7.6±0.1	0.0
	6	7.0±0.3	33.3
KY/96M	3	7.0±0.5	0.0
	6	7.2±0.5	0.0
KY/96E	3	7.3±0.4	0.0
	6	7.4±0.4	100.0
KY/99M	3	6.4±0.2	0.0
	6	5.8±0.7	0.0
KY/99E	3	7.4±0.9	0.0
	6	7.3±0.3	33.3
KY/104M	3	6.6±0.9	0.0
	6	6.9±0.5	33.3
KY/108M	3	7.5±0.6	0.0
	6	6.6±0.4	0.0
KY/108E	3	6.5±0.5	0.0
	6	6.4±0.8	33.3
KY/110M	3	5.9±0.4	0.0
	6	6.2±0.9	0.0
KY/110E	3	5.9±0.2	0.0
	6	6.8±0.7	0.0
KY/136M	3	5.3±0.3	0.0
	6	5.8±0.9	0.0
KY/136E	3	5.8±0.4	0.0
	6	5.2±0.5	0.0
KY/180M	3	6.8±0.3	0.0
	6	5.7±0.7	100.0
KY/180E	3	6.9±0.4	33.3
	6	6.4±0.1	66.7
KY/190M	3	6.8±0.5	0.0
	6	6.3±0.3	100.0
NY/18E	3	6.9±0.6	0.0
	6	6.8±1.0	33.3
CA/07E	3	7.2±0.6	0.0
	6	6.9±0.8	0.0
BN/59E	3	6.7±0.0	0.0
	6	5.9±0.1	0.0

Legend: Virus titer (log10 TCID_50_/mL at a limit of detection = 10^1.5^ TCID_50_/mL).

The mortality rates for mock- and virus-infected groups were expressed as a percentage of lethal infections (Table 2). BN/59, CA/07, KY/110 and KY/136 showed no lethality and low morbidity; whereas KY/190 and KY/180 showed the greatest lethality and substantial weight loss. Both KY/80 and NY/18 showed lethality and weight loss. On 3 and 6 days post-infection (DPI), three animals were sacrificed and the lung tissues were collected for virus titration analysis (TCID_50_ assay of lung tissue homogenate using MDCK cells). Virus titers of lungs from each mouse-infected group showed relatively little difference between the various isolates on 3 and 6 DPI (*p*>0.05 using pairwise Wilcoxon Rank-Sum test without correction for multiple comparisons, Table 2). Mice infected with egg-adapted viruses showed a higher mortality than several of the corresponding MDCK isolates (*e.g.*, see KY/96, KY/99, KY/108). However, both egg and MDCK-adapted KY/80 and KY/180 showed lethality in mice.

The influenza-specific serum IgG antibody responses in mice were measured at 6 DPI by ELISA using antigen prepared from inactivated NY/18 virus (Table 2) and reciprocal endpoints were determined. Strains KY/80E, KY/180, and KY/190 had no detectable IgG antibody by 6 DPI. The seasonal strain and all other pandemic strains produced detectable IgG antibody; the highest endpoint titers were collected from BN/59, KY/108E, NY/18E, KY99M, KY/136, and KY/110M. In some cases, these numbers reflect a small numbers of animals remaining at 6 DPI.

### Cytokine and Chemokine Responses in H1N1pdm Isolates Show Four Distinct Phenotypic Profiles

**Figure 1:**
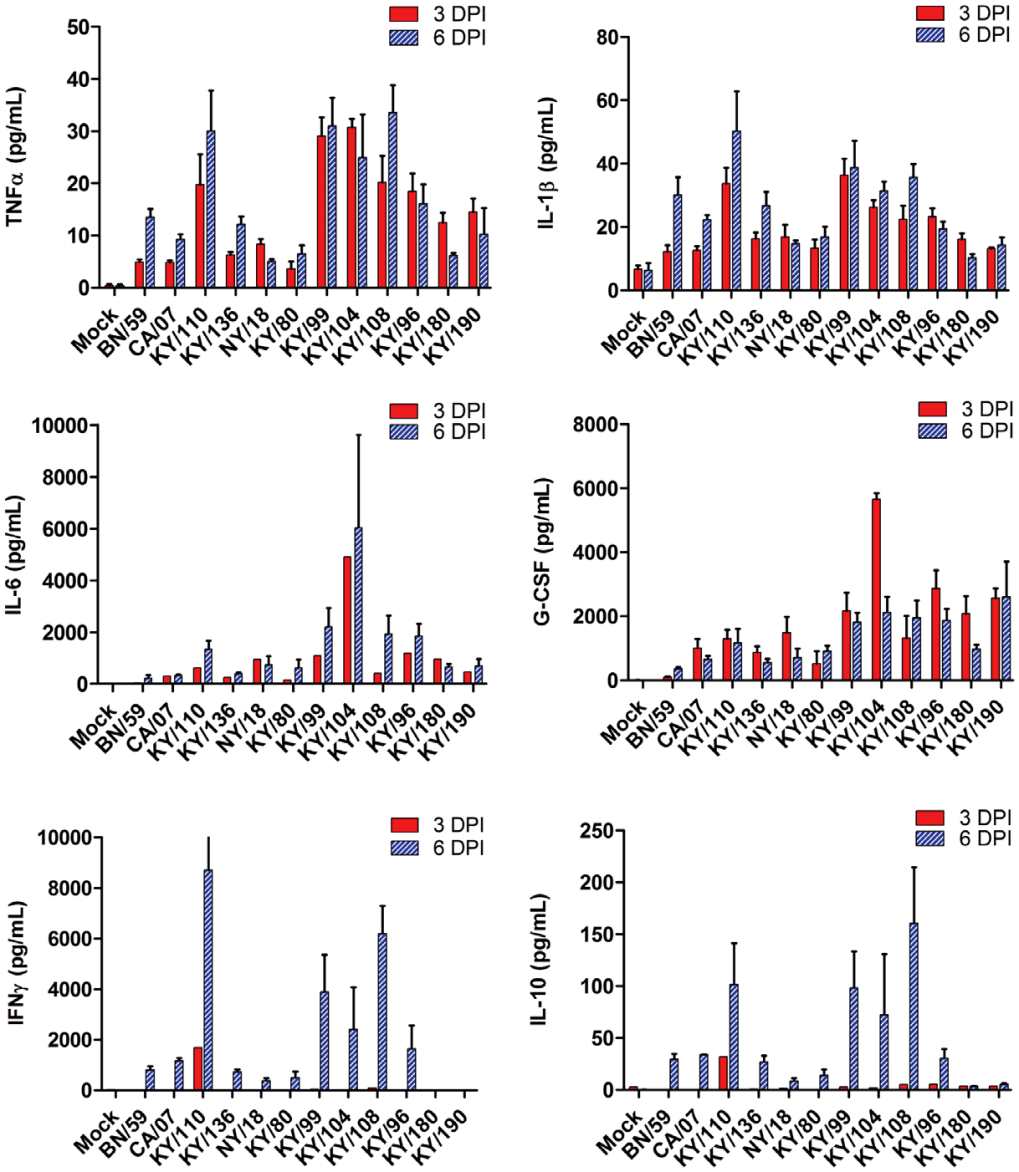
Cytokine levels in mice infected with pandemic and seasonal H1N1 influenza viruses. Six to eight week old DBA/2 mice were infected intranasally with 10^5^ TCID_50_ with a seasonal virus isolate (BN/59), Kentucky (KY/80, KY/136, KY/96, KY/99, KY/104, KY/108, KY/108, KY/110, KY/180, KY/190) or other H1N1 pandemic isolates (CA/07, NY/18) from 2009. Cytokine levels were measured at 3 and 6 DPI as described in the materials and methods and presented as mean +/− SEM (n = 6 per group, although fewer animals were available for lethal isolates).

**Figure 2:**
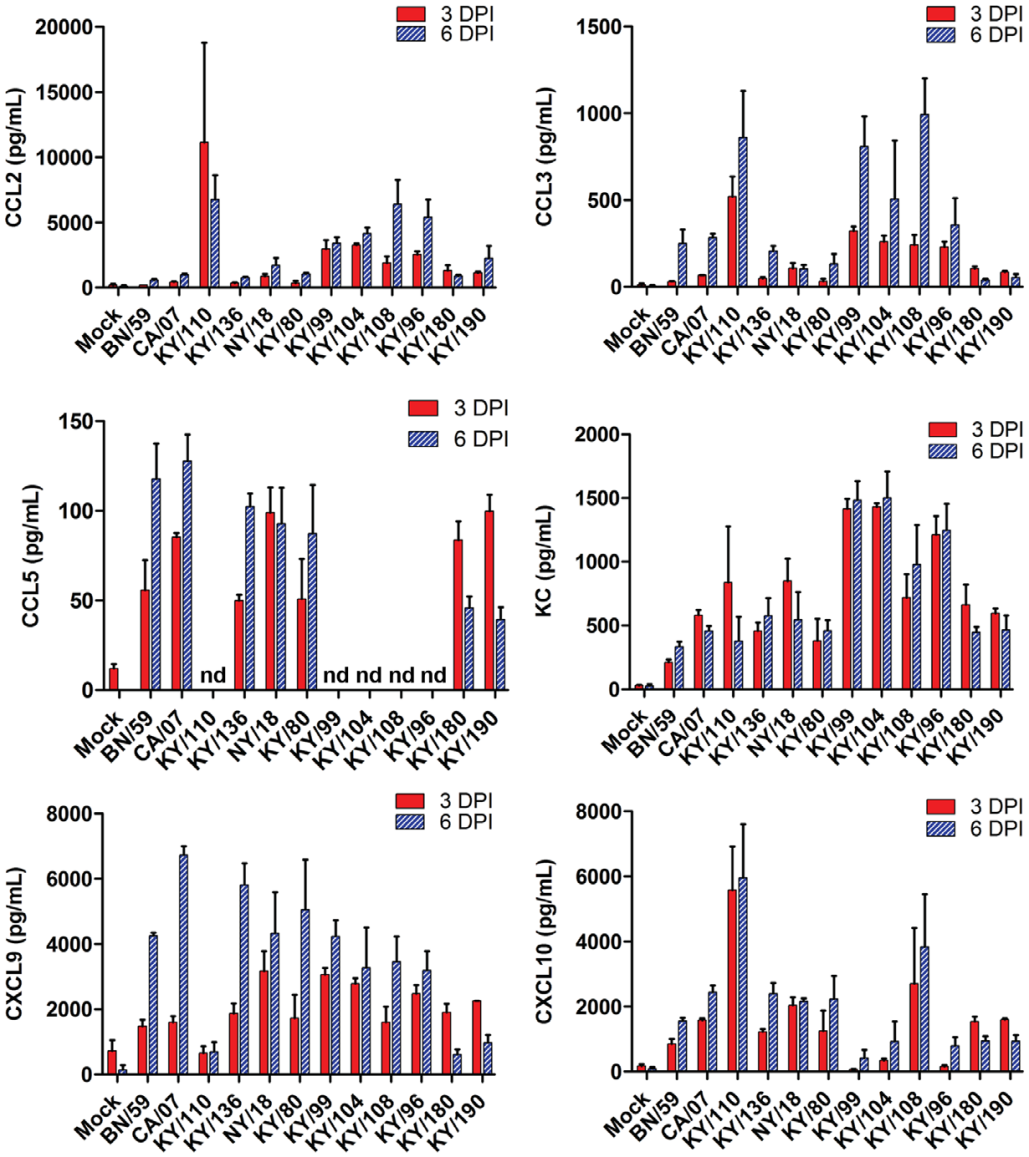
Chemokine levels in mice infected with pandemic and seasonal H1N1 influenza viruses. Six to eight week old DBA/2 mice were infected intranasally with 10^5^ TCID_50_ with a seasonal virus isolate (BN/59), Kentucky (KY/80, KY/136, KY/96, KY/99, KY/104, KY/108, KY/108, KY/110, KY/180, KY/190) or other H1N1 pandemic isolates (CA/07, NY/18) from 2009. Chemokine levels were measured at 3 and 6 DPI as described in the materials and methods and presented as mean +/− SEM (n = 6 per group when possible).

Mouse cytokine and chemokine panels were employed to gain a broad overview of the immune response at 3 and 6 DPI in the lungs and sera of mice infected with each of the viral isolates (Figures 1 and 2). No significant difference was seen between egg- and MDCK-passaged isolates; therefore, the combined data are presented in these two figures. There was an overall increase in proinflammatory cytokines (TNFα, IL-1β, IL-6 and KC) albeit different levels were noted. The levels of TNFα and IL-1β were greater in the lungs on day 3 in viral isolates with greater lethality (*e.g.*, KY/180, KY/190) as compared to those with no lethality (*e.g.*, KY/136, *p*<0.05). These same cytokines were higher in the mice with no lethality on day 6 as compared to those showing lethality. A similar temporal pattern was noted with some chemokines (*e.g.,* RANTES, Figure 2). Interferon-gamma (IFNγ) and IL-10, an anti-inflammatory cytokine, were present on day 6 in mice infected with all but the most lethal strains, KY/180, KY/190 (Figure 1, *p*<0.05).

With the exception of isolate KY/110 and KY/104, the chemokines CCL2 (MCP-1), CCL3 (MIP1α), CXCL9 (MIG), and CXCL10 (IP-10) displayed similar levels in all isolates on 3 DPI followed by a relative increase in animals infected with nonlethal versus a decrease in animals infected with lethal isolates at 6 DPI (Figure 2 and Table 2). IFNγ stimulates both IP-10 and MIG, and as expected the IFNγ levels were low in lethal isolates as compared to nonlethal isolates at 6 DPI. Cytokines and chemokines that showed limited response in mice with any viral infection included Eotaxin, GM-CSF, IL-1α, M-CSF, IL-2, IL-3, IL-4, IL-5, IL-7, IL-9, IL-12p40, IL-13, IL-15, IL-17, MIP2, LIX, MIP1β, RANTES, IL-12p70, and VEGF (data not shown).

**Figure 3:**
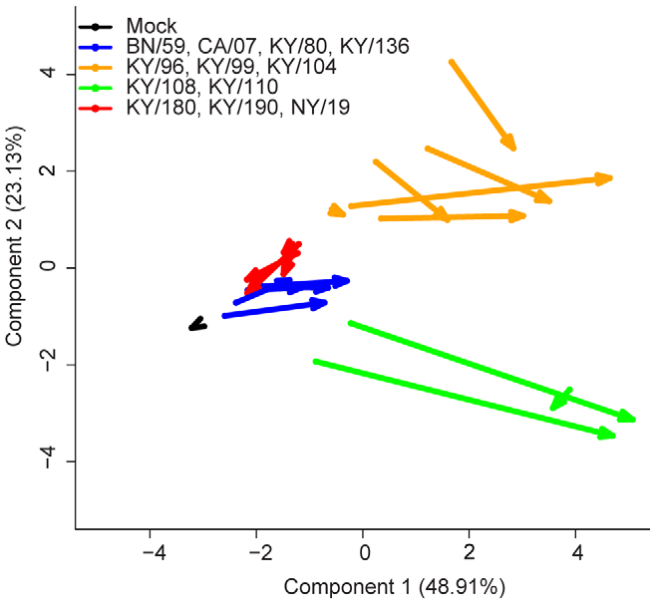
Principal components analysis of immune responses in lungs of mice to infection with pandemic and seasonal influenza viruses. A principal components analysis was performed using the 14 cytokines/chemokines analytes shown to be the most significantly different across all isolates from days 3 and 6 (determined by generalized linear model fitness testing, data not shown). The data were normalized and scaled (zero mean-centered) cytokine responses after influenza infection at 3 and 6 DPI for all viruses. The ordinate and abscissa represent the first and second components from the PCA, which explain approximately 72% of the variance. Each arrow represents the mean of a virus isolate tested in mice. Arrow tails represent day 3 components and arrow heads represent day 6 components. Using this tool to visualize the immune response, the arrows depict the trajectory of disease of the various influenza isolates as tested in DBA/2 mice. Additionally, the 12 virus isolates clustered into four distinct patterns: Group 1, BN/59, CA/07, KY/80, KY/136; Group 2, KY/96, KY/99, KY/104, KY/108; Group 3, KY/108, KY/110; Group 4, KY/180, KY/190, NY/18 (See Table S5).

A principal components analysis (PCA) was performed using 11 cytokine/chemokine measurements from the lungs of mice infected with each virus (Figure S1). The cytokines and chemokines included in the analysis were selected because they were determined to be the most significantly different between isolates on 3 and 6 DPI by generalized linear model fitness testing (data not shown). The first two components explained 72% of the variance (Figure S1) and the third component explained an additional 10% (data not shown). CCL3, TNFαα, IL-10, IL1β, and IFNγ were all highly correlated with the first component dimension (>80%), meaning these cytokines were principally important in differentiating the isolates. CXCL10, KC and G-CSF were similarly highly correlated with the second component (>70%), and CXCL9 was highly correlated with the third component dimension (83%, data not shown). The remaining variables, IL-6 and CCL2, were not highly correlated with the components used in this model, although they were significantly different between isolates on 3 and 6 DPI. The mean for each isolate on 3 and 6 DPI are plotted according to the coordinates of the first two components in Figure 3 (and Figure S1).

The method used for computing this PCA is similar to k-means cluster analysis, and therefore we expected to observe the isolates to group according to their cytokine/chemokine signatures. To better visualize these clusters, we connected each isolates’ 3 DPI coordinate to its 6 DPI coordinate with an arrow (Figure 3). According to this method of using a “time-resolved” PCA, the isolates were observed to cluster clearly into four groups representing various trajectories of disease.

The first three groups of isolates could be differentiated by their immunogenicity and low to moderate lethality. The first group includes isolates with a low lethality in mice, and was exemplified by infection with the seasonal strain BN/59, and 2009 pandemic strains CA/07, KY/80 and KY/136. The second group (KY/96, KY/99, and KY/104) included those strains with moderate lethality in the mouse model, and was similar to the first group in course of disease, but was characterized by increased inflammatory cytokines (e.g., IL-6, KC-like, and G-CSF) and chemokines (e.g., CXCL9). In contrast to the first group, these had a higher level of the anti-inflammatory cytokine IL-10 later in disease, as well as increased IFNγ at 6 DPI. The third group of influenza strains (KY/108 and KY/110) was the most immunogenic compared to the other isolates, and differed from the two groups above in that they showed the highest levels of inflammatory cytokines late in infection (e.g., IL-6, TNF-α, IL-1β) but were similar to Group 2 in terms of lethality. They showed the highest level of chemoattractant chemokines, particularly CCL2, CCL3, and CXCL10, and produced the highest levels of IL-10 and IFNγ detected in this study.

Finally, the fourth group of isolates, consisting only of KY/180, KY/190, and NY/18, were the most lethal of the viruses screened in mice. Their proinflammatory cytokines were elevated throughout the course, but not different from 3 DPI in Group 1 isolates. Most notably, these isolates failed to produce any IFNγ or IL-10 by 6 DPI. In Figure 3, the trajectory of these isolates point in an opposite direction from all other isolates, perhaps indicating a different course of disease. In summary (see Table S5), the immune responses of the virus isolates clustered similar to other phenotypic markers of virulence and lethality (Table 2).

### In-depth Comparison of the Temporal Progression of Survival, Viral Load and Immune Responses of Two Clinical Isolates, KY/136E and KY/180E, with Low and High Virulence, Respectively

**Figure 4:**
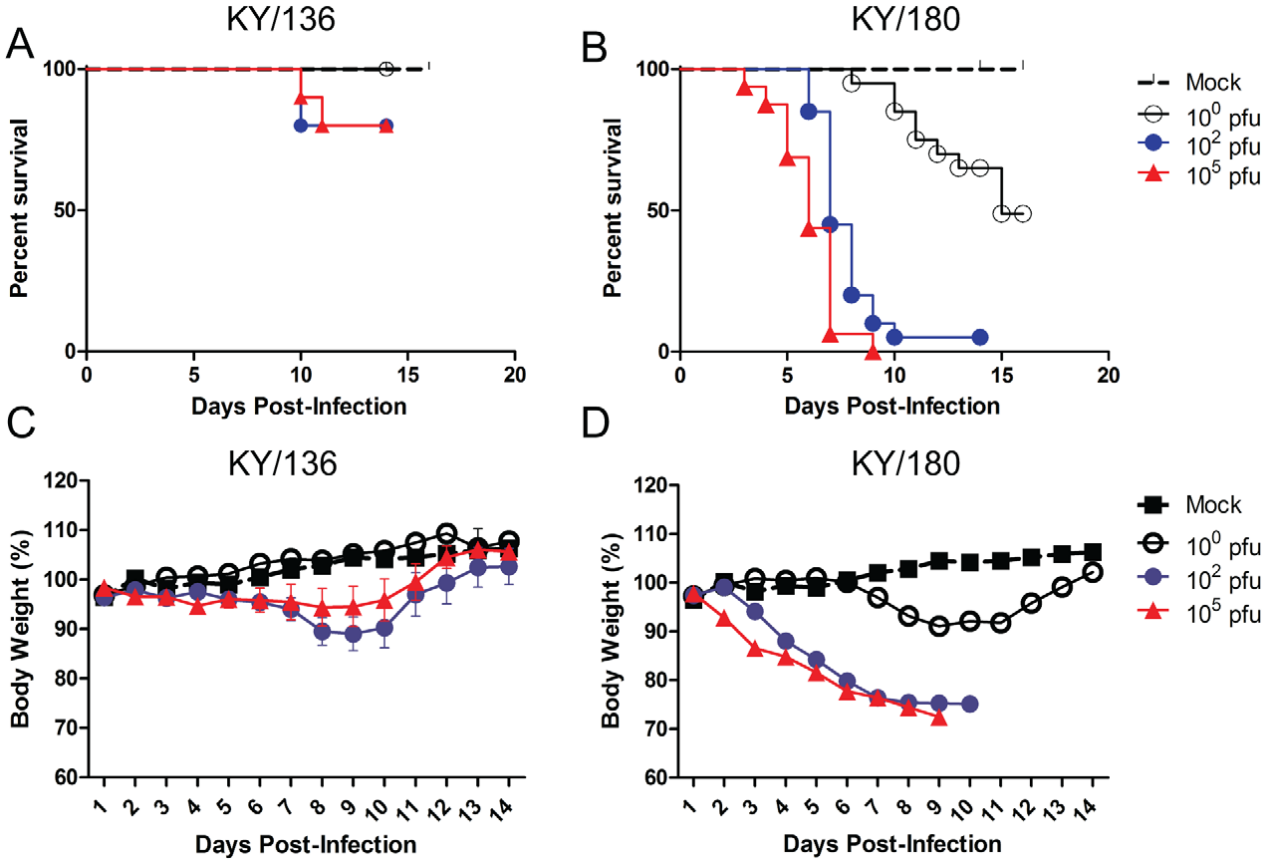
Weight loss and Kaplan-Meier curve of mice infected with KY/136 and KY/180. Mean weight change (+/− SEM) after six to eight week old DBA/2 mice were infected intranasally with 10^0^, 10^2^ and 10^5^ TCID_50_ with KY/136E (A) or KY/180E (B). Mice were examined daily for clinical signs and weighed (*n* = 10 mice per virus group). Kaplan-Meier Survival curves for these mice show KY/136E to have low lethality (C) compared to KY/180E (D).

KY/136E and KY/180E were selected from the nine H1N1pdm clinical isolates for further characterization as representatives of lower and higher virulence based on the apparent disease in mice. These isolates had similar concentrations of lung cytokines and chemokines on 3 and 6 DPI compared to other isolates and clustered together in the PCA. However, the time-resolved PCA revealed that the isolates differed in the progression of immune responses in the course of infection. To gain additional insight, we first assessed dose response to infection in DBA/2 mice. Mice were infected with 10^0^, 10^2^ or 10^5^ TCID_50_ of KY/180E or KY/136E and examined daily for clinical signs. Each day, mice were weighed and data from each dose group are presented in Figure 4A (KY/136E) and Figure 4B (KY/180E). The Kaplan-Meier survival curves of mice infected with KY/180E or KY/136E (Figure 4C and 4D, respectively) confirmed the observations reported above in terms of the general lethality of each virus, although KY/136E did show lethality in 1–2 mice on 10 DPI (Figure 4C). Mice infected with KY/180E succumbed to infection starting at 3 DPI at the high dose and on 6 DPI for the middle dose (Figure 4D). Animals were humanely euthanized upon showing a moribund state or upon a 25% loss in body weight as described in the materials and methods.

The levels of infectious virus present in the upper and lower respiratory tracts, nasal turbinates (NT) and lung, respectively, were measured at 1, 3 and 5 DPI at the three different doses (10^0^, 10^2^ or 10^5^ PFU per mouse) of KY/180E or KY/136E (Table S3). As expected, levels of virus were greatest in mice with higher doses of infection. Mice infected with KY/180E persisted at higher levels of virus over the 5 day period as compared to KY/136E which had approximately 2–3 logs lower virus in the lungs and had virus levels that decreased from 3 to 5 DPI. The infectious dose (ID_50_) and lethal dose (LD_50_) were <10^0^ TCID_50_/mouse and 10^1.2^ TCID_50_ per mouse, respectively, for KY/180E (data not shown).

**Figure 5:**
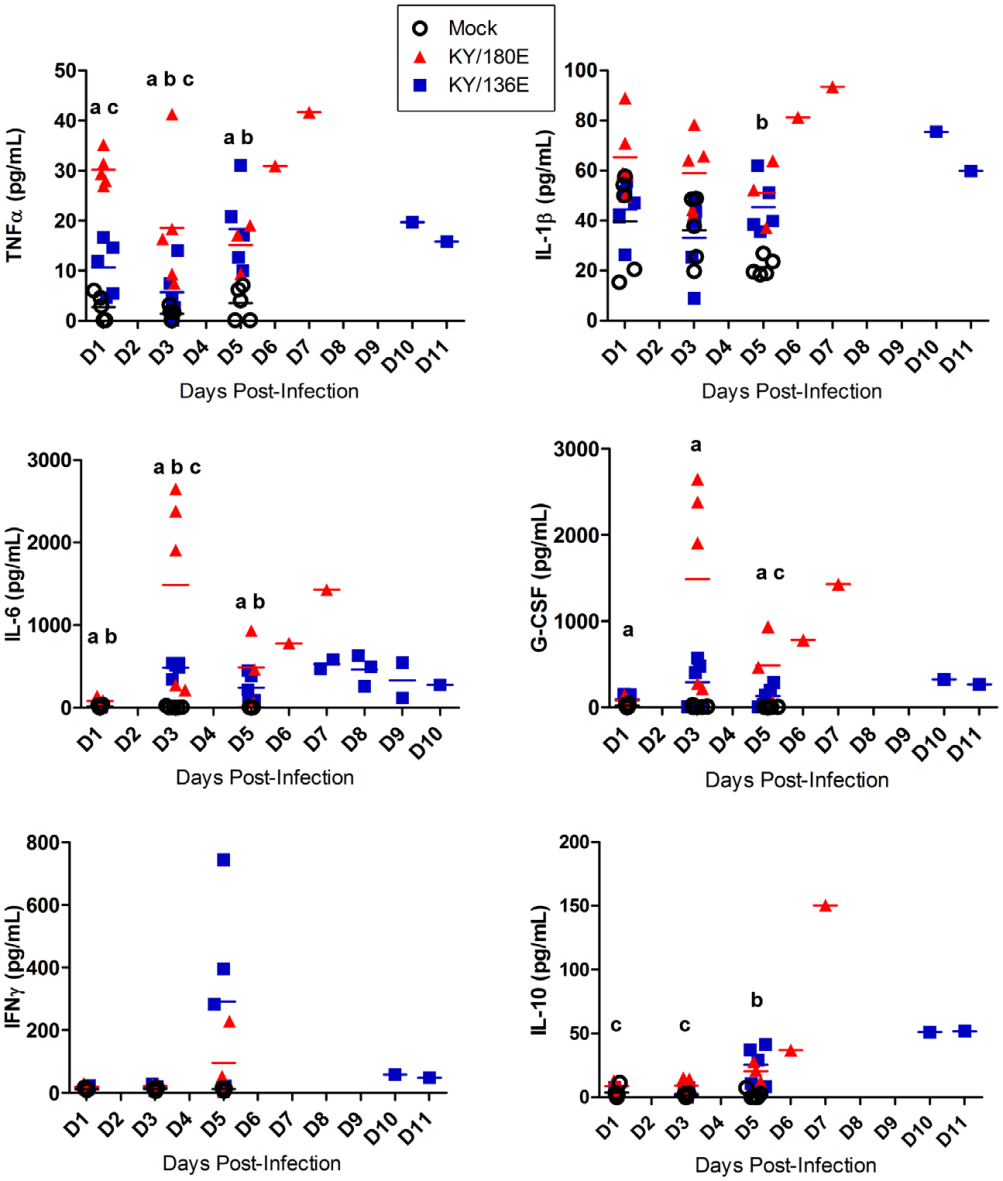
Cytokine levels in mice infected with KY/136 or KY/180. The levels of notable cytokine responses are shown for 1, 3 and 5 DPI in six to eight week old DBA/2 mice that were infected intranasally with 10^5^ TCID_50_ of KY/180E or KY/136E (*n* = 10 mice per virus group per time point). Statistical significance was determined by day using Kruskal-Wallis test followed by pairwise Wilcoxon Rank Sum *post hoc* test with Holm’s adjustment for multiple comparisons. *P*-values <0.05 are indicated by the following method: “a” = KY/180 is significantly different from mock; “b” = KY/136 is significantly different from mock; “c” = KY/180 is significantly different from KY/136.

**Figure 6:**
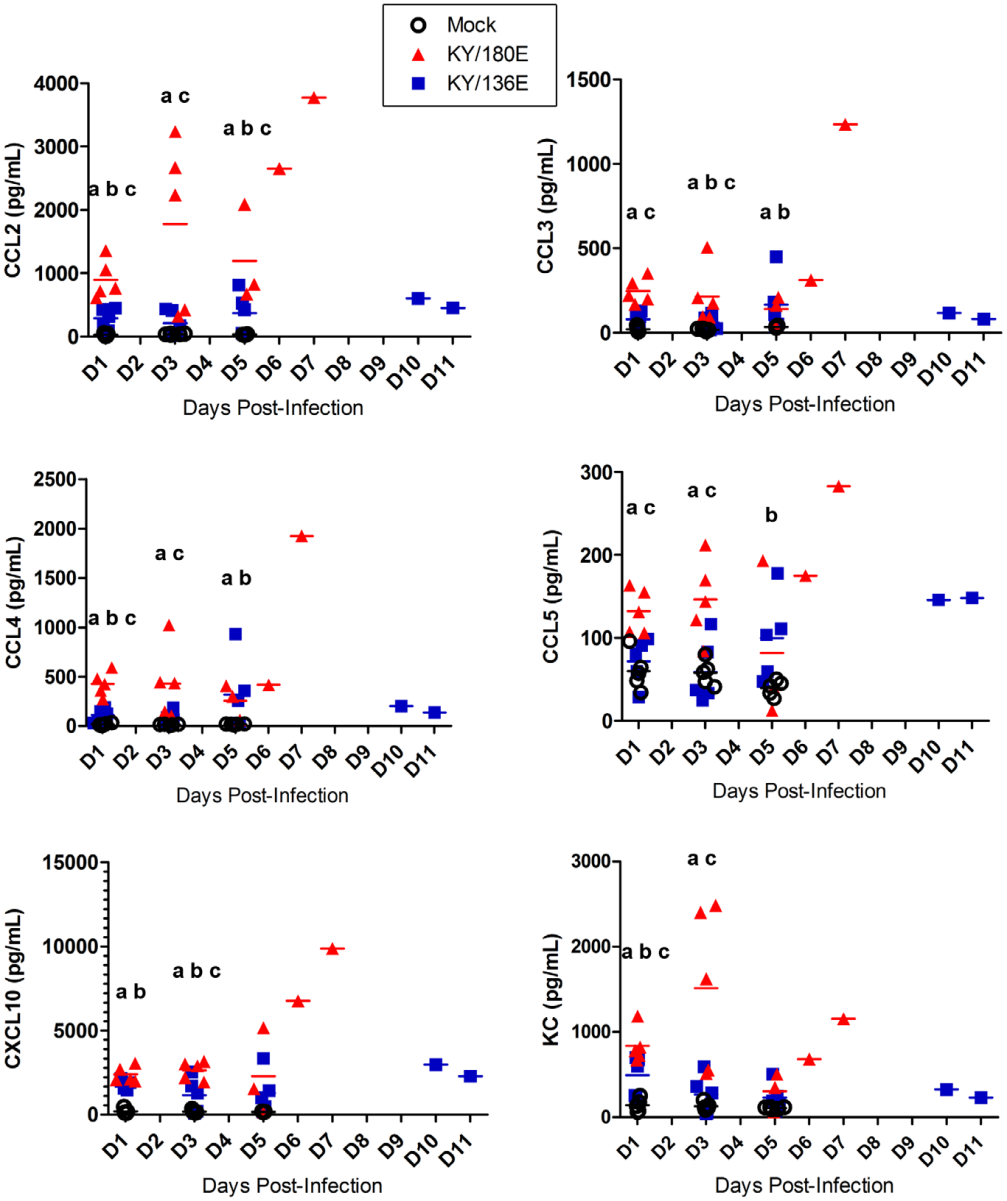
Chemokine levels in mice infected with KY/136 or KY/180. The levels of notable chemokine responses are shown for 1, 3 and 5 DPI in six to eight week old DBA/2 mice that were infected intranasally with 10^5^ TCID_50_ of KY/180E or KY/136E (*n* = 10 mice per virus group per time point). Statistical significance was determined by day using Kruskal-Wallis test followed by pairwise Wilcoxon Rank Sum *post hoc* test with Holm’s adjustment for multiple comparisons. *P*-values <0.05 are indicated by the following method: “a” = KY/180 is significantly different from mock; “b” = KY/136 is significantly different from mock; “c” = KY/180 is significantly different from KY/136.

To further dissect the immune responses of these two viruses, multiplex cytokine/chemokine bead arrays were employed to provide insight into the temporal patterns for the key cytokine and chemokine profiles noted in the broad survey of all the isolate. Mice (5 per group/per day) were infected with each of the isolates and humanely euthanized on 1, 3, and 5 DPI (Figure 5 and 6). There was an overall increase in proinflammatory cytokines (TNFα, IL-1β, IL-6 and KC) in the lungs of all mice, however, there were much higher levels of all cytokines in lungs of mice infected with KY/180E. Further the responses were much earlier and correlated with the high levels of infection noted in the lung. In contrast with KY/180E, KY/136E-infected mice showed a gradual progression in immune responses, however, the overall responses on 6 DPI of KY/136E remained low. Interferon-alpha (IFNα) and IL-12p70 levels were higher in the lungs of KY/180E-infected mice as compared to KY/136E-infected mice (Figure S2). Virus-infected mice that recovered from KY/136E infection showed a high IL-10 response by 6 DPI whereas KY/180E-infected mice showed no response (Figures 1 and 5, *p*<0.05). As expected, and also shown in Figure 1, the IFNγ levels were low in the lethal KY/180E isolate as compared to the robust response observed in the nonlethal KY/136E isolates at 6 DPI (no statistically significant difference).

**Figure 7:**
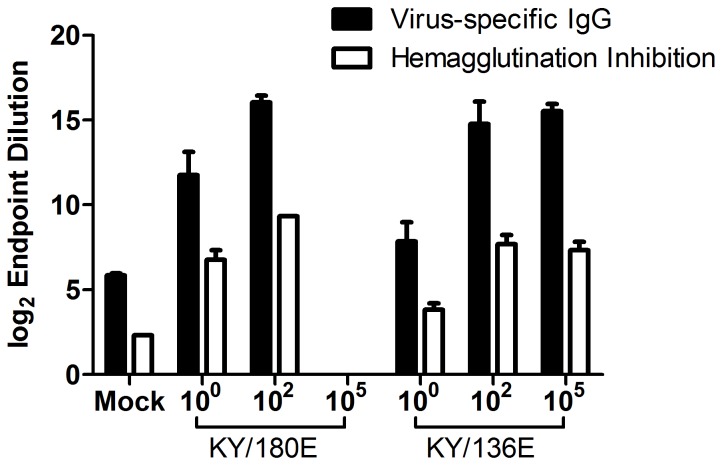
IgG responses of mice infected with different doses of KY/136 or KY/180. Serum from day 20 post-infection was taken from six to eight week old DBA/2 mice that were infected intranasally with 10^0^, 10^2^ and 10^5^ TCID_50_ of KY/180E or KY/136E (*n* = 10 mice per virus group per time point). Influenza-specific (A/NY/18/09 BPL-inactivated whole viral antigen) IgG titers were measured by ELISA and presented as average log_2_ endpoint titers (+/−SEM). No mice survived to day 20 at the high dose of KY/180. All endpoint titers for animals infected with influenza virus isolates were significantly different from mock (*p*<0.05). Endpoint titers for both IgG and HI from animals infected with KY/180 were significantly different from the same dosage amount of KY/136 (*p*<0.05).

Mice infected with the middle dose of KY/180E that survived after 6 DPI, and produced high concentrations of IFNγ, eventually became moribund with all but one euthanized by Day 10. These data suggest that the immune response differences between the two isolates are due to the high levels of infection in the lung by KY/180E. The levels of cytokines and chemokines in the lung support this observation (Figures S3–S6), showing a dose response for both isolates on 1, 3, and 5 DPI in mouse lung homogenate. Notably, a dose of 10^2^ pfu/mouse of KY/180E showed a progressive increase in chemotactic chemokines to 5DPI whereas a higher dose (10^5^ PFU/mouse) began declining in concentration of these analytes after 3DPI (Figure S3). Similar patterns were seen for proinflammatory cytokines in the lung (Figure S5) and cytokines involved in adaptive immunity initiation, IFNγ and IL12p70 (Figure S6). The endpoint titer IgG responses of the mice for the low and middle doses were similar for both viruses (Figure 7; all significantly different from control using Student’s *t*-test, *p*<0.05). Further, the HI titers were also very similar except for the lower dose, which was about 2-fold higher in mice infected with KY/180E (Figure 7).

**Figure 8:**
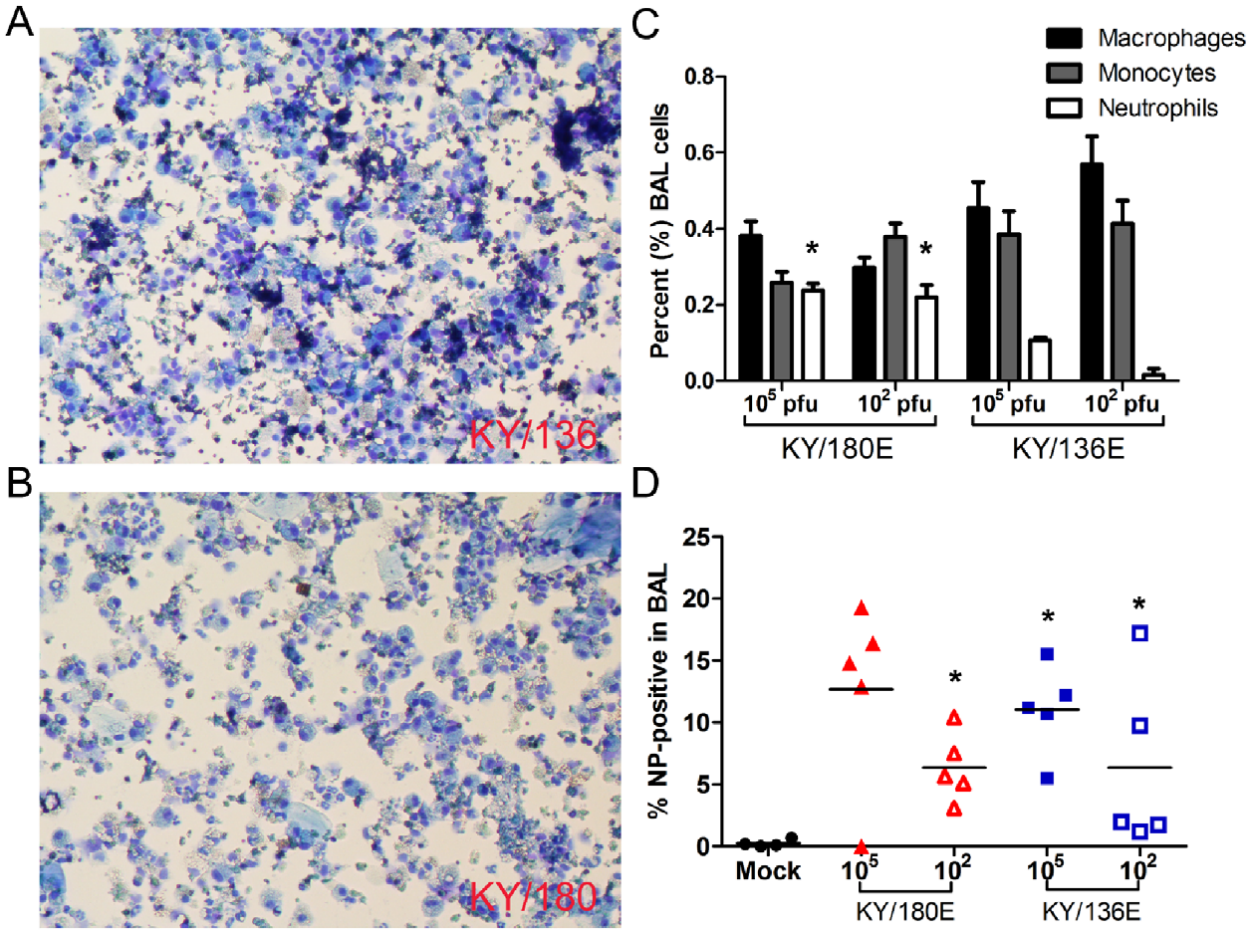
Cells within bronchial lavage fluid of mice infected with KY/136 or KY/180. The cells within the bronchoalveolar lavage fluid (BALf) at 4 DPI from DBA/2 mice infected with 10^5^ TCID_50_ of KY/136E (A) or KY/180E (B) (*n* = 5 mice per virus group per time point) were affixed to slides by cytospin centrifuge and stained (Kwik-Diff). Microscopic images from three fields per slide were counted by three blinded, independent observers. (C) The average number of macrophages, non-specific monocytes, and neutrophils are presented as a percent of the total cell count (+/− SEM). There were significantly more neutrophils in the BALf of mice infected with KY/180E virus (*p*<0.05, indicated by asterisks). (D) Cells in the BALf were fixed, permeabilized, and stained with anti-influenza nucleoprotein (NP)-FITC antibody conjugate and analyzed by flow cytometry. There were significantly more NP-positive cells in mice infected with KY/180 at a dose of 10^2^ pfu, and in mice infected with KY/136 compared to mock-infected controls (*p*<0.05, indicated by asterisks). 80% of NP-positive cells were Gr-1 positive (macrophage or neutrophil).

The leukocyte chemoattractant chemokines CCL2, CXCL10, KC (CXCL8-like), and G-CSF were greater and earlier in the lungs of mice infected with high doses of KY/180E than with a similar dose of KY/136E (Figure 6, *p*<0.05). As expected, cytospins of BAL revealed that KY/180E contained more neutrophils and monocytes on Day 4 compared to KY/136E (Figure 8A,B, *p*<0.05). Additionally, we observed a higher proportion of macrophages in the lungs in the low dose group of KY/136E compared to the higher dose group (*p*<0.05, Figure 8C). Despite there being a greater number of infiltrating leukocytes in the lungs of KY/180E-infected mice, these mice had a delayed clearance of virus in the lung.

**Figure 9:**
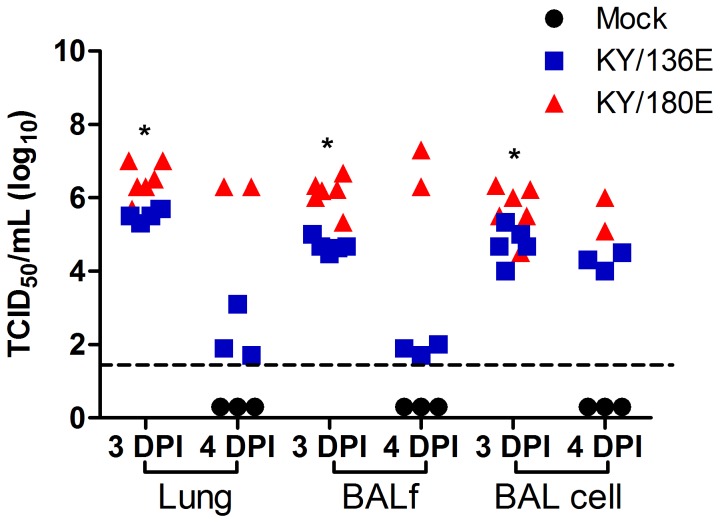
Virus titer the in lung compartments of mice infected with KY/136 or KY/180. Bronchoalveolar lavage was taken from six to nine week old DBA/2 mice that were infected with 10^5^ TCID_50_ KY/136E or KY/180E on days 3 and 4 post-infection. Cells were separated from the lavage fluid by centrifugation and the fluid (BALf), cellular (BAL cell), and whole lung homogenate were tested separately for virus by TCID_50_ assay on MDCK cells. Lung compartments taken from mice infected with KY/180 had statistically higher virus titers at 3 DPI than from mice infected with KY/136 (*p*<0.05, indicated by asterisks on the figure). It is not possible to compute statistical differences from 4 DPI, as there were only two mice infected with KY/180 in that experiment that survived to 4 DPI.

Given the apparent clearance of virus from the lungs of KY/136E (Table S4), we were interested in whether the apparent differences in lethality of the two viruses could be due to the site of virus replication. We tested virus titers from bronchoalveolar lavage fluid (BALf), from the cellular pellet of the BALc (BALc) of the mice, and from the homogenized lung after lavage using TCID_50_ assay. Samples were collected on 3 and 4 DPI because we began to see decreases in viral titer in the lungs of KY/136E-infected mice after 3DPI. In our earlier studies (Table S4), the virus titer in lung reflects lung, BALf, and BAL cells combined and we observed a nearly 3 log difference between KY/136E and KY/180E. When BAL is collected before isolation of lung tissue, we found the majority of infectious KY/136E virus present in lungs of mice was localized in the cellular fraction of the BAL (Figure 9). In contrast to KY/180E, no infectious KY/136E virus was present in the BALf by 4 DPI. Flow cytometric analysis revealed that the influenza NP-positive cells in the BAL from both isolates were also Gr1-positive (clone RB6-8C5), a marker of mouse phagocytic leukocytes, *i.e.*, Ly6C/Ly6G positive (Figure 8D).

### Infection and Replication of H1N1pdm Viruses in Macrophage Cell Lines

**Figure 10:**
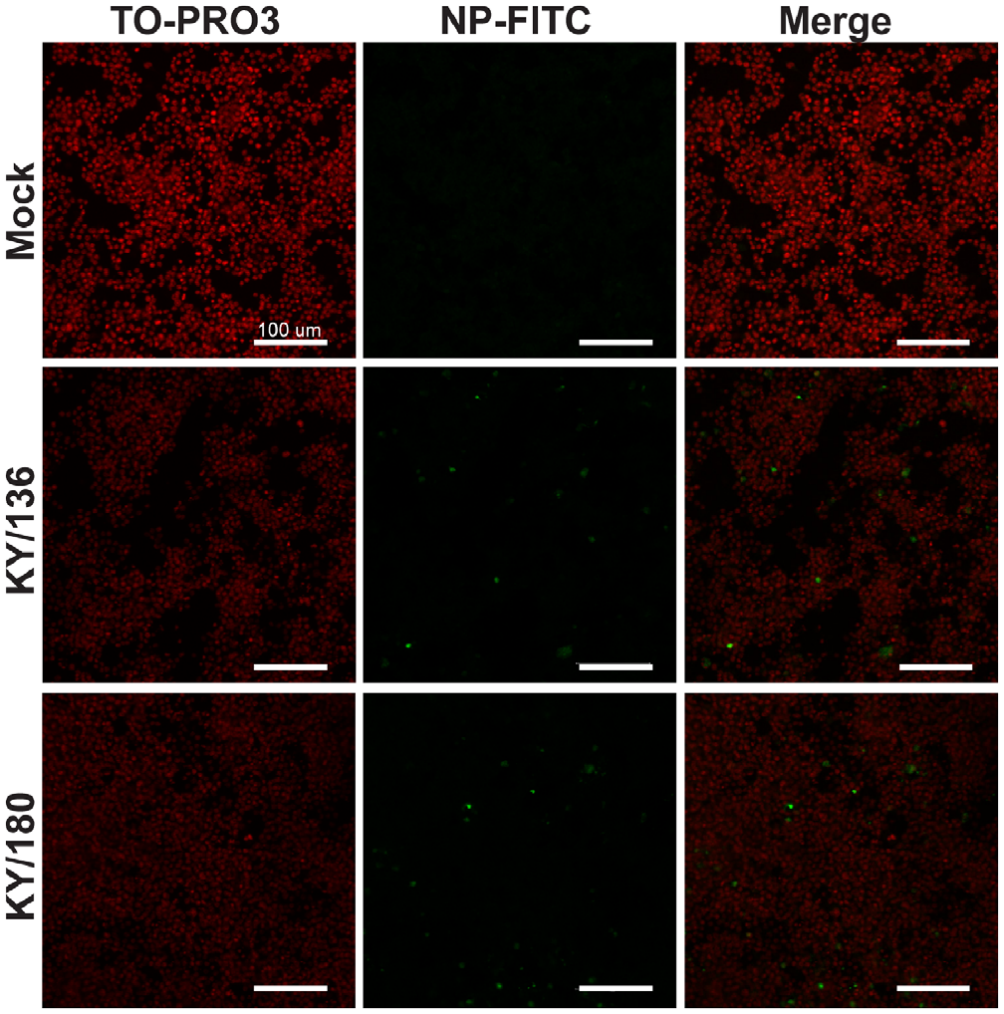
Detection of Influenza in mouse macrophage cell lines. Mouse macrophage cell line, RAW264.7, was infected *in vitro* with MOI = 1 of each influenza isolate (high pathogenic isolate, KY/180, and low pathogenic isolate, KY/136) on chambered microscopy slides. At 24 hours post-infection cells were fixed, permeabilized, and stained using a FITC antibody conjugate specific for influenza A (H1N1) nucleoprotein (green). Cells were counterstained with a nuclear dye (TO-PRO3, Molecular Probes, red), and visualized on a Zeiss LSM710 confocal microscope. Both isolates tested were observed to infect macrophages. Scale bars indicate 100 microns.

**Figure 11:**
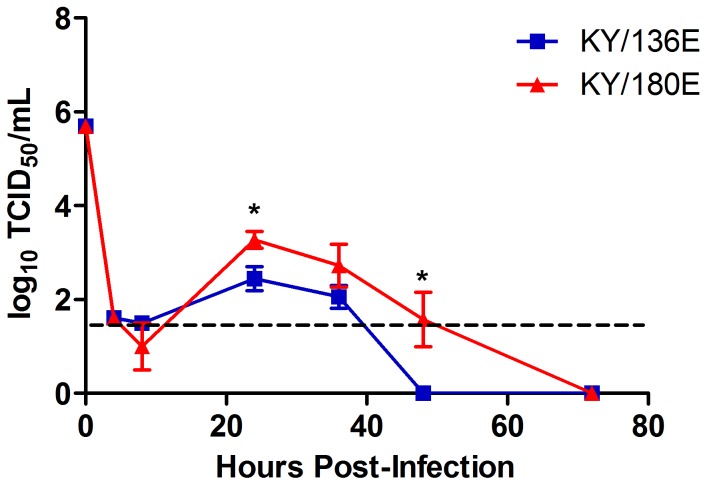
Replication of KY/180E and KY/136E in mouse macrophage cell lines. Mouse macrophage cell line, RAW264.7, was infected *in vitro* with the influenza isolates on a 24-well plate at an MOI = 1.0. After one hour of incubation, the cells were washed and returned to the incubator. Cell culture supernatants were collected over time and virus titer was determined by TCID_50_ assay. Both KY/180E and KY/136E replicate in mouse macrophage cell lines. KY/180E had significantly higher titers of virus detected at 24 and 48 hours post-infection (*p*<0.05, indicated by asterisks on the figure). Pathogenic isolate KY/180E was able to replicate better in mouse macrophages compared to the low pathogenic isolate, KY/136E.

Given the differences noted within the levels of virus in BALf and BALc, we used a BALB/c mouse macrophage cell line (RAW264.7) and a C57BL/6 mouse macrophage cell line (NR-9465) to test for the ability of KY/136E and KY/180E to infect and replicate in macrophage cells. Confocal microscopy at 24 hours post-infection showed that both viruses were able to infect macrophage cell lines (Figure 10). However, KY/180E was more efficient at replication, reaching 10-fold higher virus titers that persisted longer in both cell culture systems (Figure 11 and Figure S7). Although the cell culture supernatant was positive at 24 hours post-infection for both viruses *in vitro*, KY/180E was the only isolate detected from the cellular component in BAL (Figure 8; *p*<0.05 at 24 and 48 hours post-infection). These observations agree with our findings in the DBA/2 mouse model and suggest that KY/180 is more successful *in vivo* in replication and production of infectious virus in macrophages (Figure 8).

## Discussion

Herein we present data characterizing several isolates of H1N1pdm influenza virus taken from human patients with severe pneumonia in Kentucky, USA. Clinical surveillance from foci around the world showed that the majority of human cases of H1N1pdm were mild [26], [27]. Severe disease from H1N1pdm infection was more likely to be seen in patients with pre-existing chronic illness [28]. It was initially observed that antigenic and genetic variation in circulating pdmH1N1 2009 influenza viruses was less than what is seen during seasonal human influenza A (H1N1) [29]. Similarly, the isolates listed here show relatively low variation, differing at only 43 amino acid positions. Each isolate contained from 2 to 12 unique non-synonymous mutations from the consensus sequence.

Three of the four lethal cases occurred in western Kentucky, and 1 of these lethal cases (KY/180) contained a virus isolate with the avian-like HA RBS (G222). The D222 is most commonly associated with human H1 strains while the G222 is common in avian strains. The HA 222 position, D222G/N/S/E/Y, was a common polymorphism in severe cases of influenza in human patients, which is within the HA receptor binding site, RBS [10], [30]–[32] [33]. Additionally, the D222G has been associated with increased pathogenesis in some animal models possibly due to increased replication of the virus [30], [31], [34]–[38]. The aspartic acid functions in binding of the receptor and as a calcium antigenic site. Each of these amino acids will give rise to a specific binding affinity to the α2-6-sialic acid receptor with the D222G increasing α2-3-sialic acid receptor binding specificity [35], [37], [39]. However, it is clear that other amino acids constellations at positions 183, 186, 187, 216 and 224 will also influence these interactions [3], and may result in different overall outcomes. Additional amino acid signatures in closely related H1N1pdm isolates have also been implicated in differences in virulence in animal models. For example, in a study in a cynomolgus macaque model of two highly similar strains of H1N1pdm, A/Mexico/4487 and A/Mexico/4108, showed notable heterogeneity in virulence [40]. The amino acid variations responsible for the differences noted in these studies have not yet been reported. The HA1 of KY/180E also has a P183 (noted as 186 in [20]) rather than a S183 at the Sb antigenic site, which also has been noted to affect receptor specificity ([21]–[23]). This site, independently or together with the D222G mutation, is thought to allow binding to α-2,3 linked sialic acid residues. This substitution may allow the virus to more efficiently enter and replicate in the lower airway epithelium [23], [41]–[43].

Other mutations observed in the HA gene of the Kentucky isolates have also been reported by others. Belser, *et al.* (2010) published data that showed A/CA/04/09 also has the S83P mutation in HA (as in KY/180E), although this mutation is not a known pathogenicity determinant [12]. Ilyushina, *et al.* (2010) found the S183P (as seen in KY/180E) mutation arose from serial passage of A/CA/04/09 in mice and resulted in increased pathogenicity [21]. Xu, *et al.* (2011) report isolates from China show similar variability and share some of our mutations in HA (S83P, T203S, and V321I; seen in KY/180E, KY/96E, and KY/80E, respectively), as well as mutations in NS1 (V123I, seen in KY/99E) and PA (V14I, seen in KY/180E) [44]. Melidou *et al.* have data that suggest V321I mutation in HA may be associated with increased disease severity [24]. Finally, HA1 amino acid mutation Q293H, identified in KY/96E, has been associated with increased severity of disease observed in human cases during the pandemic [24], [25]. We include a summary of those mutations that correlate with virulence in humans and mice (Table S6). An understanding of these and other signatures associated with more virulent phenotypes will benefit insight into the biology of the virus, patient management and public health responses.

In total, KY/180E contains mutations that differ from KY/136E in seven amino acid sites in the HA protein, four sites in PA, two sites in NA, PB1 and PB2, and one site in each of M1, NS1, and NP. Comparison of the CA/07/09, NY/18, NL/602 isolates with the KY isolates showed additional variation among all isolates (Table S2). Specifically, CA/07/09, NY/18, NL/602 were similar to the KY consensus in M, HA2, PA, PB1 and PB2, In HA1, a Q223R occurred in NY/18 only. In NA, a D248N changed occurred in CA/07 and NL/602. In NS1, a D247N was noted for KY/110, CA/07, NY/18, and NL/602. Most additional changes were conserved hydrophobic changes such as Val to Iso or Met to Leu. No differences were observed among the KY, CA/07/09, NY/18, NL/602 isolates in M2 or NS2 (Table S2). Despite the small apparent genomic variation between H1N1pdm isolates, we and others have shown that there exists substantial variation in the course of disease, pathogenicity, and immune response in human patients, in animal models, and in primary cell culture [25], [40], [44]–[46]. As virulence determinants of influenza viruses typically involve the genes encoding the HA, NA, and polymerase proteins, we would expect changes in these genes to be of future interest for biological function [47]. Addition genome diversity that might give rise to unique phenotypes during infection may be revealed be deep sequencing or isolation of clones that sample the population of the virus [17].

To gain insight into the potential phenotypic variability inferred from the genotypic variability each virus was screened in DBA2 mice. All IAV tested so far, H1N1pdm, 1918 H1N1, and seasonal influenza A H1N1 virus, infect DBA2 with varying levels of lethality without adaptation [48], and express comparatively high levels of proinflammatory cytokines and chemokines [49], [50]. Herein all DBA2 mice showed high levels of infection with all isolates similar to infection of BALB/c mouse with H1N1pdm viruses [2], [12] with relatively little difference between the various isolates on 3 and 6 DPI (*p*>0.05 using pairwise Wilcoxon Rank-Sum test without correction for multiple comparisons, Table 2). However, the isolates did show three groupings in lethality (lethal, moderately lethal and not lethal) in DBA2 mice with KY/180, KY190 and KY/96 showing the highest lethality. Using a statistical method, PCA, we asked if based on these differences in lethality we might be able to cluster cytokine and chemokine responses across the H1N1pdm isolates. To our knowledge, this is the first time this method (a “time-resolved” PCA) has been used to discriminate phenotypic characteristics of viruses. Classically PCA is used as a method to reduce multivariate data so that they are better suited for a predictive statistical model. The most common contemporary usage of PCA in microbiology is during the analysis of gene expression arrays to check reproducibility of replicates. We chose to use the PCA method because of its similarity to common clustering algorithms (specifically, k-means clustering), and therefore its potential to elucidate clusters of the immune response of mice after infection with genetically similar influenza isolates. Our approach relied on two defined time points to detect differences between virus isolates using expression of multiple immune markers. Although some of the markers used in this analysis may be redundant in a virus model (*e.g.,* pro-inflammatory IL-6 and CXCL10), subtle differences that may go unnoticed are seen using this multivariate approach, particularly in the timing of these responses (and not necessarily the magnitude). Therefore, using indicators of adaptive immune priming (IFNγ) and reduction of inflammation (IL-10) were critical to the successful implementation of this strategy. More data of this kind from other influenza isolates and other respiratory virus infections may assist in the development of this method, and may reduce the number of soluble immune markers needed for analysis in a clinical setting.

Analyses of our data suggest that there are at least two main trajectories of H1N1 influenza infection in these mice: one that successfully resolves infection, and one that does not. The majority of the isolates followed a trajectory that led to resolution and clearance of the virus from the lungs. These isolates could be further clustered into three subgroups, and these subgroups differ according to the overall immunogenicity of the isolates (*i.e.,* the levels of cytokines/chemokines in the lung). In general, the isolates showing the least mortality in DBA2 mice share in common a trajectory of disease that is characterized by a gradual increase, then decrease, in inflammatory cytokines and leukocyte chemoattractant chemokines, and concomitant increase in anti-inflammatory cytokines and IFNγ. The isolates causing the highest lethality in the DBA2 mouse (KY/180E, KY/180M, and KY/190M) showed an early rise in the proinflammatory cytokines and chemokines, and a delayed or absent rise in IL-10 and IFNγ. The lethal isolates were clearly differentiated using the time-resolved PCA in that they showed a trajectory that was opposite of all other isolates. The small sample size of human patient data and their confounding comorbidities limit the inferences that can be made connecting locale, age, sex, and length of hospital stay with genotype and pathogenicity of the isolates in the human patients. Recent data suggest that human polymorphisms in genes that restrict virus infection (e.g., *IFITM3* gene) may also play an important role in outcome [51]. A final challenge is there are no relevant scoring systems for influenza pneumonia. A pneumonia severity index (PSI) is commonly used in hospitals by clinicians for community acquired pneumonia, but the comorbidities (e.g. high BMI, diabetes) complicate interpretation.

Selecting two isolates, KY/180E and KY/136E we sought to better define two of these trajectories in DBA2 mice. We initially observed KY/136E had a low level of immunogenicity in mice, which included an increase in IL-10 and IFNγ by day 6. Although mice infected with KY/136E are similar to KY/180E in concentration of IFNα, G-CSF, CCL2, KC, IL-6, CXCL10, and other inflammatory cytokines and chemokines in the lung, the isolates differ in the timing of these responses. Increases in these cytokines and chemokines are typically seen during infections with H1N1pdm influenza isolates [2], [52], [53]. For example, some studies show that IL-6 is released in response to influenza infection and that levels of IL-6 in the upper respiratory tract and in blood correlate with symptoms [54]–[56].

Type-I interferons are known to initiate the anti-viral response to influenza virus infection. One hypothesis to explain a more severe course of illness is that the virus is capable inhibiting the effect of type-I interferon signals via NS1 protein [57], [58]. The NS1 protein from KY/136E differs from KY/180E in a single amino acid at site 112 (112M versus 1121I, respectively). By day 1 post-infection, mice infected with KY/180E had a higher level of IFNα in the lung. Despite an increased IFNα and IL12-p70 response, mice infected with KY/180 failed to produce IFNγ, IL-10, and serum antibodies by 6 DPI. This most closely resembles the type of aberrant immune response seen during infections with the pandemic 1918 Influenza A (H1N1) [59], [60] and with severe seasonal influenza isolates [61]. Others have shown similar patterns of inflammation with isolates of pandemic 2009 H1N1 influenza [32], [46], [62].

Cytokines and chemokines such as CCL2 (MCP-1), CCL3 (MIP1α), CCL5 (RANTES), and CXCL10 (IP10) are responsible for activating leukocytes and attracting them to the lung compartment to clear infection [63]. Indeed, there were a higher number of leukocytes, including elevated numbers of neutrophils and monocytes, seen in the lungs of KY/180E-infected mice on days 3 and 4 post-infection. It is known that certain pathogenic H1N1 strains, such as the 1918 H1N1 strain, causes the increased neutrophil recruitment to the lung due to increased chemokine responses [64], [65], which was seen with KY/180E. Neutrophils are important for assisting in clearing influenza-infected cells from the lungs directly and indirectly [66], [67], but the precise mechanisms remain to be discovered [64], [68], [69]. Although important to the resolution of influenza virus infection, neutrophils contain cytotoxic granules that may cause severe pathology, and may contribute to morbidity and mortality [70]. An increase in neutrophils late in disease confers the pathogenicity seen in highly pathogenic strains of influenza viruses [69].

Natural Killer (NK) cells are known to be potent innate immune cells that recognize influenza hemagglutinin [71]. Classically, NK cell effector functions include release of IFNγ and direct cytotoxicity to influenza-infected cells by granule exocytosis (*e.g.,* granzyme B and perforin). Mice infected with KY/180E have higher numbers of peripheral NK cells at 3 DPI compared to KY/136E using flow cytometry (data not shown). Consistent with this finding, CCL5 (RANTES) was increased in the lungs and serum of mice infected with KY/180E compared to KY/136E (Figure 2). NK cell deficiency in mouse models of influenza infection leads to increased pathogenicity [72], [73]. Despite the increase in NK cells in KY/180E-infected mice, it has been shown that some influenza isolates may counteract this effector response, causing lysosomal degradation of the ζ chain, which is a critical component of the NK-activating receptor [74]. Additionally, DBA2 mice lack the NKG2A activating receptor that is responsible for recognizing the down-regulation of MHC-I that typically occurs with a viral infection [75]. Therefore, KY/180E may be able to exacerbate this deficiency by decreasing NK cell effector function. It has been shown that NK cells also contribute to immune pathology in response to influenza and other respiratory virus infections [76].

Finally, this study and others have shown that some strains of influenza virus are capable of infecting macrophages [77]–[80], and may alter their functionality in response to infection [81], [82]. During influenza infection, alveolar macrophages are critical in clearing virus from the lungs and are key producers of IFNγ to stimulate the adaptive immunity [64], [65], [76], [83]–[88]. Upon infection, influenza viruses may interfere with their normal function [86], [89], [90]. Of the two isolates tested here, mice infected with KY/136E showed clearance of virus from the lung by day 4. [86], [89], [90]. Our *in vitro* data using mouse macrophage cell lines indicates that KY/180E was able to produce infectious virus better than KY/136E. Additionally, mice infected with KY/180E had a higher number of Gr-1 positive infiltrating cells into the lungs by day 4, a delayed IL-10 production, and very low levels of IFNγresulting in a deficient virus clearance. Both our *in vivo* and *in vitro* data show that KY/180E can infect macrophages, which may affect the cell’s ability to release IFNγ and explain the increase pathogenicity observed with this virus compared to KY/136E. Future studies will focus on understanding the mechanism(s) involved in the virus-induced deregulation of macrophage function *in vitro* and *in vivo*. Understanding the genetic basis for the differences in the interaction between the virus and macrophages as well as other pulmonary and immune cells will contribute to understanding the course of influenza infection in patients, and assist in predicting potential virulence during future outbreaks.

## Materials and Methods

### Detection of Influenza in Clinical Nasopharyngeal Swab Specimens

Nasal swab samples were provided by the SIPS project. A nasopharyngeal swab was taken from all patients meeting the case definition, which was defined as a patient admitted to an intensive care unit with the physician diagnosis of community-acquired pneumonia. The University of Louisville Institutional Review Board Human Subject Protection Program Office (HSPPO) approved this study prior to any data collection (#08.0399). Informed consent was waived because it was a retrospective chart review and the data were analyzed anonymously. The nasopharyngeal swabs were taken as part of ‘standard of care’ diagnosis. Detection of influenza virus was accomplished using the Respiratory Viral Panel (RVP) detection kit (Luminex Corp., Austin, TX), recently approved for *in vitro* diagnostics by the FDA. The RVP is a reverse-transcriptase, real-time PCR assay which is multiplexed to detect 12 viral targets in a single reaction well. Those targets include Influenza A, Influenza A-subtype H1, Influenza A-subtype H3, Influenza B, RSV-A, RSV-B, Parainflunza 1, Parainfluenza 2, Parainfluenza 3, human metapneumovirus, Rhinovirus and Adenovirus. Nasal swabs were collected from each patient and placed in Universal Transport Media (Copan Diagnostics, Inc, Murietta, CA). Nucleic acid was extracted using the QIAmp Mini-Elute Viral Spin Kit® (Qiagen, Valencia, CA) into a final volume of 50.0 µl. A 5.0 µl aliquot was combined with 20 µl of RVP mastermix and processed through several rounds of amplification, including a single-tube multiplex reverse-transcriptase PCR, followed by a multiplex target-specific primer extension protocol, a bead-hybridization step, and finally a data acquisition step in the Luminex reader. Results are qualitative and read-outs are Positive, Negative or a No Call (equivalent).

### Viruses and Virus Isolation

The viruses CA/07, NY/18, and BN/59 were kindly provided by the Centers for Disease Control and Prevention, Virus Surveillance and Diagnosis Branch, Influenza Division. Viruses from clinical cases from KY present in nasopharyngeal swab specimens were isolated and propagated in the chorioallantoic cavity of 10-day-old embryonated chicken eggs (Charles River) and MDCK cell line purchased from ATCC (CCL-34). The allantoic fluid containing infectious particles or MDCK supernate was harvested 72 h after inoculation. MDCK cells were infected in DMEM virus culture medium (DMEM containing 0.2% BSA, 1% PEN/STREP, and 2 µg/mL of Trypsin-TPCK). The infectious virus titer of the resulting seed stock was determined by TCID_50_ and the titer calculated by Reed and Muench [91], and confirmed by plaque assay on MDCK cells. Egg passage E2 was used for the studies involving KY/180E and KY/136E reported herein.

### RNA Isolation and Sequencing

To sequence each of the gene segments of each H1N1pdm isolate, we followed the method described by Inoue et al [92] with minor modifications. In brief, total RNA was isolated with MagMAX AI/ND Viral RNA isolation kit (Ambion) from each virus seed stock solutions. Five µL of RNA was used to synthesize cDNA with SuperScriptase III (Invitrogen) using the FWuni12 and RVuni13 primers based on those reported by Inoue et al [92] and the cDNAs were PCR-amplified based with Accuprime (Invitrogen) The amplified products were separated by agarose gel electrophoresis and the DNA bands corresponding to the size of each segment were purified using the Wizard SV gel Clean-Up System (Promega). The purified DNAs were used as templates for automated dideoxy sequencing with BigDye 3.1 cycle sequencing kit (Applied Biosystems). To sequence the entire gene we used approximately 500 bp overlapping gene-specific primers in both directions. For the sequencing of HA gene, we used FWuni12, RVuni13, HA462_f (5′-GACTCGAACAAAGGTGTAACGG-3′) and HA1202_r (5′-GTCAATGGCATTCTGTGTGCTC-3′) as sequencing primers. For the sequencing of NA gene, we used FWuni12, RVuni13, NA_521r (5′-TGACCAAGCGACTGACTCAA-3′), NA376_f (5′-CCCTTGGAATGCAGAACCTT-3′) and NA905_f (5′-CGTGGGTGTCTTTCAACCAGAA-3′). The sequences were assembled with the SeqScape™ program (Applied Biosystems). Alignments of nucleic and amino acid sequences were completed to identify polymorphisms with other H1N1pdm isolates and sequences for all isolates were submitted through the Influenza Research Database and uploaded into the GenBank (accession numbers provided in Table S4).

### Ethics Statement for Animal Studies

Mice studies were approved by the University of Louisville Institutional Animal Care and Use Committee with Veterinary Medicine tasked to monitor and support all animal experiments. Research was conducted in compliance with the Animal Welfare Act and other federal statutes and regulations relating to animals and experiments involving animals and adheres to principles stated in the *Guide for the Care and Use of Laboratory Animals*, National Research Council, 1996. The facilities where this research was conducted in a fully accredited by the Association for Assessment and Accreditation of Laboratory Animal Care International.

### Mice Studies

Six to nine-week-old female DBA/2 mice were purchased from Jackson Laboratories (Bar Harbor, ME) and housed in the vivarium managed by UofL at the Research Resource Center or at the Regional Biocontainment Laboratory. The mice received food and water *ad libitum,* and all experiments were conducted in accordance with rules of the Institutional Animal Control and Use Committee of UofL. Mice were anaesthetized by isofluorane inhalation and infected intranasally with in a total volume of 30 µl at the dose as outlined in the figure legends and text. Viruses were adjusted to the dose required in PBS (pH 7.2). Animals were observed daily for morbidity and measured for weight loss. All mice showing more than 25% body weight loss were considered to have reached the experimental end point and were euthanized humanely.

Mice were euthanized on the days noted in the results and on figure legends and blood, nasal turbinates and lung tissues were collected and stored appropriately until analyses. For collection of BAL after euthanasia, the neck and thoracic area were disinfected by soaking with 70% ethanol, and the trachea exposed by dissection. An 18G catheter needle was inserted into upper trachea, the needle removed, and the outer catheter sheath moved into the lower trachea. A 3 mL syringe filled with 1 mL of DPBS was used to carefully inject DPBS into the lungs, and aspirate BAL fluid. Collected fluids were transferred to an ice-cold, sterile 15 mL conical tube on ice and repeated once more as above. BAL fluids were immediately centrifuged at 500g for 10 minutes to separate fluids and cells. After centrifugation, supernatants are separated and kept separately at −80°C freezer until used for TCID_50_ assay. Cells pellets were washed once by adding 2 mL ice-cold DPBS and centrifuged again at 500*g* for 10 min. Cells were resuspended in 1 mL DPBS and kept at −80°C until used.

### TCID_50_ Assay for Viral Load in Tissues and Bronchial Lavage Fluid

Tissues were homogenized in ice-cold virus culture medium (DMEM containing 0.2% BSA, 1% PEN/STREP) within a biosafety cabinet. Tissue homogenates were clarified by centrifugation (4000*g* for 20 min at 4°C) prior to storage at −80°C, and later analyzed by TCID_50_ or Luminex cytometric bead array (next section). For measurement of the level of infectious virus present in tissue samples, the titers of the virus were determined by TCID_50_ assay by titration of the clarified tissue homogenates or BALf on MDCK cells. The limit of virus detection was typically 10^1.5^ TCID_50_/ml or as indicated in each figure. Virus titers were calculated by the method of Reed and Muench [91], and are expressed as the mean log_10_ TCID_50_ per milliliter. Tissues in which no virus was detected were given a value of 10^1.0^ TCID_50_/ml for calculation of the mean titer.

### Quantification of Cytokine and Chemokine Levels in Lungs

Sera and lungs from mice euthanized on days as noted in figure legends and text were analyzed by using Luminex® xMAP® technology-based assay kit (Millipore) according to the manufacturer’s protocol. The final reaction plate was read with a Luminex 100 or FlexMAP 3D machine and specific concentrations were calculated from a standard using Luminex xPONENT software.

### Statistics and PCA

R (version 2.13.0) base statistical package and GraphPad software package were used in the analysis of data and generation of figures. Generalized linear models were constructed using each analyte included in the initial screen of Kentucky isolates in DBA/2 mice as a response variable (cytokine/chemokine concentrations) and isolate, DPI, and egg vs. MDCK stock, were used as predictors. The stock designation of the virus was removed from the model after it was seen that it did not contribute to explain significant proportion of the variance. Log likelihood ratios were used to compare the relative fit of each model, and the top 12 models showing the highest parameter estimates for the isolates were chosen for inclusion into the PCA (*i.e.,* the cytokines/chemokines that were the most different between isolates, controlling for DPI, selected from the best-fit models). The R package “FactoMineR” (version 1.14) was used to perform the PCA. Statistically significant differences between multiple groups was assessed using Kruskal-Wallis tests followed by *post hoc* tests using pairwise Wilcoxon Rank Sum tests unless otherwise noted. Multiple comparisons were adjusted according to Holm’s method and a *p*-value <0.05 is considered significant.

### Cytospin and Flow Cytometry

BAL cells were centrifuged at 300g for 5 min and washed twice in PBS. Cells were resuspended in 0.4 mL PBS and loaded into Shandon Cytospin™ centrifuge funnels. The cytocentrifuge chambers were spun at 1000 rpm for 5 min. Slides were air dried in a BSC and then were fixed and stained with Eosin and Methylene Blue (Kwik-Diff, Thermo Scientific). Slides were inspected under a light microscope.

For flow cytometry analysis, BAL cells were washed in PBS as above and stained with antibodies specific to mouse CD3 (500A2 Pacific Blue, BD Biosciences), Gr-1 (RB6-8C5 phycoerythrin, eBioscience), and CD49b (DX5 phycoerythrin-Cy7, eBioscience). Cells were then washed and fixed in 4% paraformaldehyde for 15 minutes at room temperature, followed by permeabilization with 0.2% saponin in 2% BSA for 20 minutes. Cells were then stained intracellularly for influenza nucleoprotein using a fluorescein isothiocyanate conjugated antibody (NP-FITC, AbCam #ab20921) for 30 minutes. Cells were washed and analyzed on a FACSAria II flow cytometer (BD).

### Mouse Macrophages Experiments

RAW264.7 cells (ATCC #TIB-71) were seeded onto glass chamber slides (LabTek) and allowed to rest overnight. The cells were gently washed in PBS and virus was added at 1.0 MOI. The cells were washed and fixed at 24 hours post-infection and permeabilized with 0.2% saponin in 10% FBS. Cells were stained for intracellular influenza nucleoprotein as above, and a nuclear counter stain was included for the final 10 minutes (TO-PRO3, Molecular Probes). Cells were washed, mounted under a coverslip with ProLong Antifade media (Invitrogen), and visualized using a Zeiss LSM/710 confocal microscope. Kinetics of infection were determined by infecting mouse macrophage cell lines, RAW264.7 or BEI Resources #NR9456 (NIAID, NIH), in triplicate wells of a 24-well plate in DMEM supplemented with 0.2% BSA, 1% Pen/Strep, 2.5% L-glutamine, and 25 mM HEPES. Supernatants were taken and centrifuged prior to performing a TCID50 assay using MDCK cells. Virus titers were confirmed at 24 hours using a plaque assay.

## Supporting Information

Figure S1 **Principal Components Analysis (PCA) of mouse lung cytokine and chemokine expression after challenge with clinical Influenza A (H1N1) virus isolates from Kentucky, 2009.** Standardized mean values for each cytokine/chemokine are plotted from Day 3 and Day 6 post-infection (n = 3, each) against the first two principal components, accounting for 72% of the variation in the analysis.
(TIF)

Figure S2 **Cytokine and chemokine profiles from mouse lung homogenate.** DBA/2 mice were infected with KY/180E, KY/136E (10^5^ pfu), or mock-infected with PBS. The mice were sacrificed 1, 3, and 5 days post-infection (*n* = 5 mice per group-day). Samples from moribund mice taken after Day 5 post-infection were also analyzed when available. Bars indicate mean concentration.
(TIF)

Figure S3 (TIF)

Figure S4 **Dose response of chemokines in the lungs of mice infected with KY/136 or KY/180 influenza A (H1N1) virus isolates.** Mice were infected with 10^0^, 10^2^, or 10^5^ pfu of virus and samples were collected upon euthanasia on days 1, 3, or 5 post-challenge (D1, D3, and D5, respectively). *n* = 5 mice per dose-day for all groups except for mice infected with 10^5^ pfu of KY/180 where only 2/5 mice survived to D5.
(TIF)

Figure S5 **Dose response of cytokines in the lungs of mice infected with KY/136 or KY/180 influenza A (H1N1) virus isolates.** Mice were infected with 10^0^, 10^2^, or 10^5^ pfu of virus and samples were collected upon euthanasia on days 1, 3, or 5 post-challenge (D1, D3, and D5, respectively). *n* = 5 mice per dose-day for all groups except for mice infected with 10^5^ pfu of KY/180 where only 2/5 mice survived to D5.
(TIF)

Figure S6 **Dose response of cytokines in the lungs of mice infected with KY/136 or KY/180 influenza A (H1N1) virus isolates.** Mice were infected with 10^0^, 10^2^, or 10^5^ pfu of virus and samples were collected upon euthanasia on days 1, 3, or 5 post-challenge (D1, D3, and D5, respectively). *n* = 5 mice per dose-day for all groups except for mice infected with 10^5^ pfu of KY/180 where only 2/5 mice survived to D5.
(TIF)

Figure S7 **Replication kinetics of KY/180E and KY/180E isolates in C57BL/6 mouse macrophages.** The macrophage cell line, BEIR #NR-9465, was infected at 1.0 MOI (two independent experiments at *n* = 3 per experiment) and clarified supernatants were taken at 4, 24, 48, and 72 hour post-infection. Virus titers were measured by TCID_50_ assay on MDCK cells.
(TIF)

Table S1 **Comorbidities associated with severe, hospitalized influenza pneumonia patients.**
(DOCX)

Table S2 **Variants noted in amino acid sequence alignments of H1N1pdm clinical isolates.**
(DOCX)

Table S3 **GenBank accession numbers of H1N1pdm isolates.**
(DOCX)

Table S4 Virus titers (TCID_50_/ml*) on 1, 3, and 5 days post-infection in lungs and nasal turbinates of DBA/2 mice infected with KY/180E and KY/136E.
(DOCX)

Table S5 **Relative magnitude of immune responses of H1N1pdm isolates in mice in groups revealed by principal component analysis clustering.**
(DOCX)

Table S6 **Summary of references to mutations in Influenza A (H1N1) isolates with observed virulence.**
(DOCX)

## References

[1] Peiris JS, Poon LL, Guan Y (2009) Emergence of a novel swine-origin influenza A virus (S-OIV) H1N1 virus in humans. J Clin Virol 45: 169–173. 19540800 10.1016/j.jcv.2009.06.006 PMC4894826

[2] Itoh Y, Shinya K, Kiso M, Watanabe T, Sakoda Y, et al (2009) In vitro and in vivo characterization of new swine-origin H1N1 influenza viruses. Nature 460: 1021–1025. 19672242 10.1038/nature08260 PMC2748827

[3] Maines TR, Jayaraman A, Belser JA, Wadford DA, Pappas C, et al (2009) Transmission and pathogenesis of swine-origin 2009 A(H1N1) influenza viruses in ferrets and mice. Science 325: 484–487. 19574347 10.1126/science.1177238 PMC2953552

[4] Van Kerkhove MD, Vandemaele KA, Shinde V, Jaramillo-Gutierrez G, Koukounari A, et al (2011) Risk Factors for Severe Outcomes following 2009 Influenza A (H1N1) Infection: A Global Pooled Analysis. PLoS Med 8: e1001053. 21750667 10.1371/journal.pmed.1001053 PMC3130021

[5] Louie JK, Jean C, Acosta M, Samuel MC, Matyas BT, et al (2011) A review of adult mortality due to 2009 pandemic (H1N1) influenza A in California. PLoS One 6: e18221. 21483677 10.1371/journal.pone.0018221 PMC3071719

[6] Lucker LM, Kherad O, Iten A, Wagner N, Descombes M, et al (2011) Clinical features and outcome of hospitalised adults and children with the 2009 influenza A H1N1 infection at Geneva’s University Hospital. Swiss Med Wkly 141: w13177. 21416409 10.4414/smw.2011.13177

[7] Kuster SP, Drews S, Green K, Blair J, Davis I, et al (2010) Epidemiology of influenza-associated hospitalization in adults, Toronto, 2007/8. Eur J Clin Microbiol Infect Dis 29: 835–843. 20428910 10.1007/s10096-010-0935-x PMC2889286

[8] Shieh WJ, Blau DM, Denison AM, Deleon-Carnes M, Adem P, et al (2010) 2009 pandemic influenza A (H1N1): pathology and pathogenesis of 100 fatal cases in the United States. Am J Pathol 177: 166–175. 20508031 10.2353/ajpath.2010.100115 PMC2893660

[9] Gill JR, Sheng ZM, Ely SF, Guinee DG, Beasley MB, et al (2010) Pulmonary pathologic findings of fatal 2009 pandemic influenza A/H1N1 viral infections. Arch Pathol Lab Med 134: 235–243. 20121613 10.5858/134.2.235 PMC2819217

[10] Wang B, Dwyer DE, Soedjono M, Shi H, Matlho K, et al (2011) Evidence of the circulation of pandemic influenza (H1N1) 2009 with D222D/G/N/S hemagglutinin polymorphisms during the first wave of the 2009 influenza pandemic. J Clin Virol 52: 304–306. 21925936 10.1016/j.jcv.2011.08.023

[11] Fezeu L, Julia C, Henegar A, Bitu J, Hu FB, et al (2011) Obesity is associated with higher risk of intensive care unit admission and death in influenza A (H1N1) patients: a systematic review and meta-analysis. Obes Rev 12: 653–659. 21457180 10.1111/j.1467-789X.2011.00864.x

[12] Belser JA, Wadford DA, Pappas C, Gustin KM, Maines TR, et al (2010) Pathogenesis of pandemic influenza A (H1N1) and triple-reassortant swine influenza A (H1) viruses in mice. J Virol 84: 4194–4203. 20181710 10.1128/JVI.02742-09 PMC2863721

[13] Wong SS, Yuen KY (2006) Avian influenza virus infections in humans. Chest 129: 156–168. 16424427 10.1378/chest.129.1.156 PMC7094746

[14] Woo PC, Tung ET, Chan KH, Lau CC, Lau SK, et al (2010) Cytokine profiles induced by the novel swine-origin influenza A/H1N1 virus: implications for treatment strategies. J Infect Dis 201: 346–353. 20030555 10.1086/649785 PMC7202468

[15] Medina RA, Garcia-Sastre A (2011) Influenza A viruses: new research developments. Nat Rev Microbiol 9: 590–603. 21747392 10.1038/nrmicro2613 PMC10433403

[16] Baillie GJ, Galiano M, Agapow PM, Myers R, Chiam R, et al (2012) Evolutionary dynamics of local pandemic H1N1/2009 influenza virus lineages revealed by whole-genome analysis. J Virol 86: 11–18. 22013031 10.1128/JVI.05347-11 PMC3255882

[17] Ghedin E, Laplante J, DePasse J, Wentworth DE, Santos RP, et al (2011) Deep sequencing reveals mixed infection with 2009 pandemic influenza A (H1N1) virus strains and the emergence of oseltamivir resistance. J Infect Dis 203: 168–174. 21288815 10.1093/infdis/jiq040 PMC3071067

[18] Ozawa M, Basnet S, Burley LM, Neumann G, Hatta M, et al (2011) Impact of amino acid mutations in PB2, PB1-F2, and NS1 on the replication and pathogenicity of pandemic (H1N1) 2009 influenza viruses. J Virol 85: 4596–4601. 21325408 10.1128/JVI.00029-11 PMC3126221

[19] Kao CL, Chan TC, Tsai CH, Chu KY, Chuang SF, et al (2012) Emerged HA and NA mutants of the pandemic influenza H1N1 viruses with increasing epidemiological significance in Taipei and Kaohsiung, Taiwan, 2009–10. PLoS One 7: e31162. 22328930 10.1371/journal.pone.0031162 PMC3273476

[20] Reid AH, Janczewski TA, Lourens RM, Elliot AJ, Daniels RS, et al (2003) 1918 influenza pandemic caused by highly conserved viruses with two receptor-binding variants. Emerg Infect Dis 9: 1249–1253. 14609459 10.3201/eid0910.020789 PMC3033089

[21] Ilyushina NA, Khalenkov AM, Seiler JP, Forrest HL, Bovin NV, et al (2010) Adaptation of pandemic H1N1 influenza viruses in mice. Journal of virology 84: 8607–8616. 20592084 10.1128/JVI.00159-10 PMC2918990

[22] Ye J, Sorrell EM, Cai Y, Shao H, Xu K, et al (2010) Variations in the hemagglutinin of the 2009 H1N1 pandemic virus: potential for strains with altered virulence phenotype? PLoS pathogens 6: e1001145. 20976194 10.1371/journal.ppat.1001145 PMC2954835

[23] Suphaphiphat P, Franti M, Hekele A, Lilja A, Spencer T, et al (2010) Mutations at positions 186 and 194 in the HA gene of the 2009 H1N1 pandemic influenza virus improve replication in cell culture and eggs. Virology journal 7: 157. 20630098 10.1186/1743-422X-7-157 PMC2914672

[24] Melidou A, Gioula G, Exindari M, Chatzidimitriou D, Diza E, et al (2010) Molecular and phylogenetic analysis of the haemagglutinin gene of pandemic influenza H1N1 2009 viruses associated with severe and fatal infections. Virus research 151: 192–199. 20493216 10.1016/j.virusres.2010.05.005

[25] Glinsky GV (2010) Genomic analysis of pandemic (H1N1) 2009 reveals association of increasing disease severity with emergence of novel hemagglutinin mutations. Cell cycle 9: 958–970. 20160492 10.4161/cc.9.5.10913

[26] Blair PJ, Wierzba TF, Touch S, Vonthanak S, Xu X, et al (2010) Influenza epidemiology and characterization of influenza viruses in patients seeking treatment for acute fever in Cambodia. Epidemiology and infection 138: 199–209. 19698213 10.1017/S095026880999063X

[27] Cao B, Li XW, Mao Y, Wang J, Lu HZ, et al (2009) Clinical features of the initial cases of 2009 pandemic influenza A (H1N1) virus infection in China. The New England journal of medicine 361: 2507–2517. 20007555 10.1056/NEJMoa0906612

[28] Van Kerkhove MD, Vandemaele KA, Shinde V, Jaramillo-Gutierrez G, Koukounari A, et al (2011) Risk factors for severe outcomes following 2009 influenza A (H1N1) infection: a global pooled analysis. PLoS medicine 8: e1001053. 21750667 10.1371/journal.pmed.1001053 PMC3130021

[29] Garten RJ, Davis CT, Russell CA, Shu B, Lindstrom S, et al (2009) Antigenic and genetic characteristics of swine-origin 2009 A(H1N1) influenza viruses circulating in humans. Science 325: 197–201. 19465683 10.1126/science.1176225 PMC3250984

[30] Kilander A, Rykkvin R, Dudman SG, Hungnes O (2010) Observed association between the HA1 mutation D222G in the 2009 pandemic influenza A(H1N1) virus and severe clinical outcome, Norway 2009–2010. Euro Surveill 15. 10.2807/ese.15.09.19498-en 20214869

[31] Mak GC, Au KW, Tai LS, Chuang KC, Cheng KC, et al. (2010) Association of D222G substitution in haemagglutinin of 2009 pandemic influenza A (H1N1) with severe disease. Euro Surveill 15. 20394715

[32] Berdal JE, Mollnes TE, Waehre T, Olstad OK, Halvorsen B, et al (2011) Excessive innate immune response and mutant D222G/N in severe A (H1N1) pandemic influenza. The Journal of infection 63: 308–316. 21781987 10.1016/j.jinf.2011.07.004

[33] Chan PK, Lee N, Joynt GM, Choi KW, Cheung JL, et al (2011) Clinical and virological course of infection with haemagglutinin D222G mutant strain of 2009 pandemic influenza A (H1N1) virus. J Clin Virol 50: 320–324. 21330192 10.1016/j.jcv.2011.01.013

[34] Chutinimitkul S, Herfst S, Steel J, Lowen AC, Ye J, et al (2010) Virulence-associated substitution D222G in the hemagglutinin of 2009 pandemic influenza A(H1N1) virus affects receptor binding. Journal of virology 84: 11802–11813. 20844044 10.1128/JVI.01136-10 PMC2977876

[35] Belser JA, Jayaraman A, Raman R, Pappas C, Zeng H, et al (2011) Effect of D222G Mutation in the Hemagglutinin Protein on Receptor Binding, Pathogenesis and Transmissibility of the 2009 Pandemic H1N1 Influenza Virus. PLoS One 6: e25091. 21966421 10.1371/journal.pone.0025091 PMC3178596

[36] Anton A, Marcos MA, Martinez MJ, Ramon S, Martinez A, et al (2010) D225G mutation in the hemagglutinin protein found in 3 severe cases of 2009 pandemic influenza A (H1N1) in Spain. Diagn Microbiol Infect Dis 67: 207–208. 20356695 10.1016/j.diagmicrobio.2010.02.002

[37] Liu Y, Childs RA, Matrosovich T, Wharton S, Palma AS, et al (2010) Altered receptor specificity and cell tropism of D222G hemagglutinin mutants isolated from fatal cases of pandemic A(H1N1) 2009 influenza virus. J Virol 84: 12069–12074. 20826688 10.1128/JVI.01639-10 PMC2977873

[38] Abed Y, Pizzorno A, Hamelin ME, Leung A, Joubert P, et al (2011) The 2009 pandemic H1N1 D222G hemagglutinin mutation alters receptor specificity and increases virulence in mice but not in ferrets. The Journal of infectious diseases 204: 1008–1016. 21881115 10.1093/infdis/jir483

[39] Xu R, McBride R, Nycholat CM, Paulson JC, Wilson IA (2012) Structural characterization of the hemagglutinin receptor specificity from the 2009 H1N1 influenza pandemic. Journal of virology 86: 982–990. 22072785 10.1128/JVI.06322-11 PMC3255799

[40] Safronetz D, Rockx B, Feldmann F, Belisle SE, Palermo RE, et al (2011) Pandemic swine-origin H1N1 influenza A virus isolates show heterogeneous virulence in macaques. Journal of virology 85: 1214–1223. 21084481 10.1128/JVI.01848-10 PMC3020514

[41] Tumpey TM, Maines TR, Van Hoeven N, Glaser L, Solorzano A, et al (2007) A two-amino acid change in the hemagglutinin of the 1918 influenza virus abolishes transmission. Science 315: 655–659. 17272724 10.1126/science.1136212

[42] Takemae N, Ruttanapumma R, Parchariyanon S, Yoneyama S, Hayashi T, et al (2010) Alterations in receptor-binding properties of swine influenza viruses of the H1 subtype after isolation in embryonated chicken eggs. The Journal of general virology 91: 938–948. 20007353 10.1099/vir.0.016691-0

[43] Watanabe T, Shinya K, Watanabe S, Imai M, Hatta M, et al (2011) Avian-type receptor-binding ability can increase influenza virus pathogenicity in macaques. Journal of virology 85: 13195–13203. 21937653 10.1128/JVI.00859-11 PMC3233164

[44] Xu L, Bao L, Zhou J, Wang D, Deng W, et al (2011) Genomic polymorphism of the pandemic A (H1N1) influenza viruses correlates with viral replication, virulence, and pathogenicity in vitro and in vivo. PLoS One 6: e20698. 21698272 10.1371/journal.pone.0020698 PMC3115934

[45] Patel JR, Vora KP, Tripathi S, Zeng H, Tumpey TM, et al (2011) Infection of lung epithelial cells with pandemic 2009 A(H1N1) influenza viruses reveals isolate-specific differences in infectivity and host cellular responses. Viral immunology 24: 89–99. 21449719 10.1089/vim.2010.0122

[46] Meunier I, Embury-Hyatt C, Stebner S, Gray M, Bastien N, et al (2012) Virulence differences of closely related pandemic 2009 H1N1 isolates correlate with increased inflammatory responses in ferrets. Virology 422: 125–131. 22074911 10.1016/j.virol.2011.10.018

[47] Pappas C, Aguilar PV, Basler CF, Solorzano A, Zeng H, et al (2008) Single gene reassortants identify a critical role for PB1, HA, and NA in the high virulence of the 1918 pandemic influenza virus. Proceedings of the National Academy of Sciences of the United States of America 105: 3064–3069. 18287069 10.1073/pnas.0711815105 PMC2268585

[48] Pica N, Iyer A, Ramos I, Bouvier NM, Fernandez-Sesma A, et al (2011) The DBA.2 mouse is susceptible to disease following infection with a broad, but limited, range of influenza A and B viruses. Journal of virology 85: 12825–12829. 21917963 10.1128/JVI.05930-11 PMC3209355

[49] Srivastava B, Blazejewska P, Hessmann M, Bruder D, Geffers R, et al (2009) Host genetic background strongly influences the response to influenza a virus infections. PLoS One 4: e4857. 19293935 10.1371/journal.pone.0004857 PMC2654507

[50] Trammell RA, Liberati TA, Toth LA (2012) Host genetic background and the innate inflammatory response of lung to influenza virus. Microbes and infection/Institut Pasteur 14: 50–58. 10.1016/j.micinf.2011.08.008 21920449

[51] Everitt AR, Clare S, Pertel T, John SP, Wash RS, et al (2012) IFITM3 restricts the morbidity and mortality associated with influenza. Nature 484: 519–523. 22446628 10.1038/nature10921 PMC3648786

[52] Hagau N, Slavcovici A, Gonganau DN, Oltean S, Dirzu DS, et al (2010) Clinical aspects and cytokine response in severe H1N1 influenza A virus infection. Crit Care 14: R203. 21062445 10.1186/cc9324 PMC3220006

[53] Lee N, Chan PK, Wong CK, Wong KT, Choi KW, et al (2011) Viral clearance and inflammatory response patterns in adults hospitalized for pandemic 2009 influenza A(H1N1) virus pneumonia. Antivir Ther 16: 237–247. 21447873 10.3851/IMP1722

[54] Hayden FG, Treanor JJ, Fritz RS, Lobo M, Betts RF, et al (1999) Use of the oral neuraminidase inhibitor oseltamivir in experimental human influenza: randomized controlled trials for prevention and treatment. JAMA 282: 1240–1246. 10517426 10.1001/jama.282.13.1240

[55] Fritz RS, Hayden FG, Calfee DP, Cass LM, Peng AW, et al (1999) Nasal cytokine and chemokine responses in experimental influenza A virus infection: results of a placebo-controlled trial of intravenous zanamivir treatment. J Infect Dis 180: 586–593. 10438343 10.1086/314938

[56] Kaiser L, Fritz RS, Straus SE, Gubareva L, Hayden FG (2001) Symptom pathogenesis during acute influenza: interleukin-6 and other cytokine responses. J Med Virol 64: 262–268. 11424113 10.1002/jmv.1045

[57] Garcia-Sastre A (2011) Induction and evasion of type I interferon responses by influenza viruses. Virus Res 162: 12–18. 22027189 10.1016/j.virusres.2011.10.017 PMC3640439

[58] Fernandez-Sesma A, Marukian S, Ebersole BJ, Kaminski D, Park MS, et al (2006) Influenza virus evades innate and adaptive immunity via the NS1 protein. J Virol 80: 6295–6304. 16775317 10.1128/JVI.02381-05 PMC1488970

[59] Kobasa D, Takada A, Shinya K, Hatta M, Halfmann P, et al (2004) Enhanced virulence of influenza A viruses with the haemagglutinin of the 1918 pandemic virus. Nature 431: 703–707. 15470432 10.1038/nature02951

[60] Baskin CR, Bielefeldt-Ohmann H, Tumpey TM, Sabourin PJ, Long JP, et al (2009) Early and sustained innate immune response defines pathology and death in nonhuman primates infected by highly pathogenic influenza virus. Proc Natl Acad Sci U S A 106: 3455–3460. 19218453 10.1073/pnas.0813234106 PMC2642661

[61] Svitek N, Rudd PA, Obojes K, Pillet S, von Messling V (2008) Severe seasonal influenza in ferrets correlates with reduced interferon and increased IL-6 induction. Virology 376: 53–59. 18420248 10.1016/j.virol.2008.02.035

[62] To KK, Hung IF, Li IW, Lee KL, Koo CK, et al (2010) Delayed clearance of viral load and marked cytokine activation in severe cases of pandemic H1N1 2009 influenza virus infection. Clinical infectious diseases : an official publication of the Infectious Diseases Society of America 50: 850–859. 20136415 10.1086/650581 PMC7107930

[63] Herold S, von Wulffen W, Steinmueller M, Pleschka S, Kuziel WA, et al (2006) Alveolar epithelial cells direct monocyte transepithelial migration upon influenza virus infection: impact of chemokines and adhesion molecules. J Immunol 177: 1817–1824. 16849492 10.4049/jimmunol.177.3.1817

[64] Tumpey TM, Garcia-Sastre A, Taubenberger JK, Palese P, Swayne DE, et al (2005) Pathogenicity of influenza viruses with genes from the 1918 pandemic virus: functional roles of alveolar macrophages and neutrophils in limiting virus replication and mortality in mice. Journal of virology 79: 14933–14944. 16282492 10.1128/JVI.79.23.14933-14944.2005 PMC1287592

[65] Perrone LA, Plowden JK, Garcia-Sastre A, Katz JM, Tumpey TM (2008) H5N1 and 1918 pandemic influenza virus infection results in early and excessive infiltration of macrophages and neutrophils in the lungs of mice. PLoS pathogens 4: e1000115. 18670648 10.1371/journal.ppat.1000115 PMC2483250

[66] Tate MD, Brooks AG, Reading PC, Mintern JD (2012) Neutrophils sustain effective CD8(+) T-cell responses in the respiratory tract following influenza infection. Immunology and cell biology 90: 197–205. 21483446 10.1038/icb.2011.26

[67] Hashimoto Y, Moki T, Takizawa T, Shiratsuchi A, Nakanishi Y (2007) Evidence for phagocytosis of influenza virus-infected, apoptotic cells by neutrophils and macrophages in mice. Journal of immunology 178: 2448–2457. 10.4049/jimmunol.178.4.2448 17277152

[68] Fujisawa H (2008) Neutrophils play an essential role in cooperation with antibody in both protection against and recovery from pulmonary infection with influenza virus in mice. J Virol 82: 2772–2783. 18184718 10.1128/JVI.01210-07 PMC2258992

[69] Tate MD, Ioannidis LJ, Croker B, Brown LE, Brooks AG, et al (2011) The role of neutrophils during mild and severe influenza virus infections of mice. PLoS One 6: e17618. 21423798 10.1371/journal.pone.0017618 PMC3056712

[70] Tate MD, Deng YM, Jones JE, Anderson GP, Brooks AG, et al (2009) Neutrophils ameliorate lung injury and the development of severe disease during influenza infection. J Immunol 183: 7441–7450. 19917678 10.4049/jimmunol.0902497

[71] Stein-Streilein J, Bennett M, Mann D, Kumar V (1983) Natural killer cells in mouse lung: surface phenotype, target preference, and response to local influenza virus infection. Journal of immunology 131: 2699–2704. 6644021

[72] Stein-Streilein J, Guffee J, Fan W (1988) Locally and systemically derived natural killer cells participate in defense against intranasally inoculated influenza virus. Regional immunology 1: 100–105. 3275212

[73] Stein-Streilein J, Guffee J (1986) In vivo treatment of mice and hamsters with antibodies to asialo GM1 increases morbidity and mortality to pulmonary influenza infection. Journal of immunology 136: 1435–1441. 3944461

[74] Mao H, Tu W, Liu Y, Qin G, Zheng J, et al (2010) Inhibition of human natural killer cell activity by influenza virions and hemagglutinin. Journal of virology 84: 4148–4157. 20164232 10.1128/JVI.02340-09 PMC2863726

[75] Vance RE, Jamieson AM, Cado D, Raulet DH (2002) Implications of CD94 deficiency and monoallelic NKG2A expression for natural killer cell development and repertoire formation. Proceedings of the National Academy of Sciences of the United States of America 99: 868–873. 11782535 10.1073/pnas.022500599 PMC117397

[76] Abdul-Careem MF, Mian MF, Yue G, Gillgrass A, Chenoweth MJ, et al (2012) Critical role of natural killer cells in lung immunopathology during influenza infection in mice. The Journal of infectious diseases 206: 167–177. 22561366 10.1093/infdis/jis340

[77] Reading PC, Whitney PG, Pickett DL, Tate MD, Brooks AG (2010) Influenza viruses differ in ability to infect macrophages and to induce a local inflammatory response following intraperitoneal injection of mice. Immunology and cell biology 88: 641–650. 20142836 10.1038/icb.2010.11

[78] Reading PC, Miller JL, Anders EM (2000) Involvement of the mannose receptor in infection of macrophages by influenza virus. Journal of virology 74: 5190–5197. 10799594 10.1128/jvi.74.11.5190-5197.2000 PMC110872

[79] Hofmann P, Sprenger H, Kaufmann A, Bender A, Hasse C, et al (1997) Susceptibility of mononuclear phagocytes to influenza A virus infection and possible role in the antiviral response. Journal of leukocyte biology 61: 408–414. 9103226 10.1002/jlb.61.4.408

[80] Weinheimer VK, Becher A, Tonnies M, Holland G, Knepper J, et al. (2012) Influenza A Viruses Target Type II Pneumocytes in the Human Lung. J Infect Dis. 10.1093/infdis/jis455 PMC7107318 22829640

[81] Osterlund P, Pirhonen J, Ikonen N, Ronkko E, Strengell M, et al (2010) Pandemic H1N1 2009 influenza A virus induces weak cytokine responses in human macrophages and dendritic cells and is highly sensitive to the antiviral actions of interferons. Journal of virology 84: 1414–1422. 19939920 10.1128/JVI.01619-09 PMC2812319

[82] Josset L, Belser JA, Pantin-Jackwood MJ, Chang JH, Chang ST, et al (2012) Implication of inflammatory macrophages, nuclear receptors, and interferon regulatory factors in increased virulence of pandemic 2009 H1N1 influenza A virus after host adaptation. J Virol 86: 7192–7206. 22532695 10.1128/JVI.00563-12 PMC3416346

[83] Tate MD, Pickett DL, van Rooijen N, Brooks AG, Reading PC (2010) Critical role of airway macrophages in modulating disease severity during influenza virus infection of mice. Journal of virology 84: 7569–7580. 20504924 10.1128/JVI.00291-10 PMC2897615

[84] Wijburg OL, DiNatale S, Vadolas J, van Rooijen N, Strugnell RA (1997) Alveolar macrophages regulate the induction of primary cytotoxic T-lymphocyte responses during influenza virus infection. Journal of virology 71: 9450–9457. 9371606 10.1128/jvi.71.12.9450-9457.1997 PMC230250

[85] Peschke T, Bender A, Nain M, Gemsa D (1993) Role of macrophage cytokines in influenza A virus infections. Immunobiology 189: 340–355. 8125516 10.1016/s0171-2985(11)80365-7

[86] Hui KP, Lee SM, Cheung CY, Ng IH, Poon LL, et al (2009) Induction of proinflammatory cytokines in primary human macrophages by influenza A virus (H5N1) is selectively regulated by IFN regulatory factor 3 and p38 MAPK. J Immunol 182: 1088–1098. 19124752 10.4049/jimmunol.182.2.1088

[87] Kim HM, Lee YW, Lee KJ, Kim HS, Cho SW, et al (2008) Alveolar macrophages are indispensable for controlling influenza viruses in lungs of pigs. J Virol 82: 4265–4274. 18287245 10.1128/JVI.02602-07 PMC2293066

[88] Seo SH, Webby R, Webster RG (2004) No apoptotic deaths and different levels of inductions of inflammatory cytokines in alveolar macrophages infected with influenza viruses. Virology 329: 270–279. 15518807 10.1016/j.virol.2004.08.019

[89] Sakabe S, Iwatsuki-Horimoto K, Takano R, Nidom CA, Le MQ, et al (2011) Cytokine production by primary human macrophages infected with highly pathogenic H5N1 or pandemic H1N1 2009 influenza viruses. J Gen Virol 92: 1428–1434. 21367984 10.1099/vir.0.030346-0 PMC3168279

[90] van Riel D, Leijten LM, van der Eerden M, Hoogsteden HC, Boven LA, et al (2011) Highly pathogenic avian influenza virus H5N1 infects alveolar macrophages without virus production or excessive TNF-alpha induction. PLoS Pathog 7: e1002099. 21731493 10.1371/journal.ppat.1002099 PMC3121882

[91] Reed LJ, Muench H (1938) A simple method of estimating fifty percent endpoints. American Journal of Hygiene 27: 493–497.

[92] Inoue E, Wang X, Osawa Y, Okazaki K (2010) Full genomic amplification and subtyping of influenza A virus using a single set of universal primers. Microbiol Immunol 54: 129–134. 20236422 10.1111/j.1348-0421.2009.00193.x

