# Supplementary figures and images for: Phenotypic Differences in Virulence and Immune Response in Closely Related Clinical Isolates of Influenza A 2009 H1N1 Pandemic Viruses in Mice
Source: PLoS One. 2013 Feb 18;8(2):e56602. doi: 10.1371/journal.pone.0056602 (PMC3575477; doi:10.1371/journal.pone.0056602)

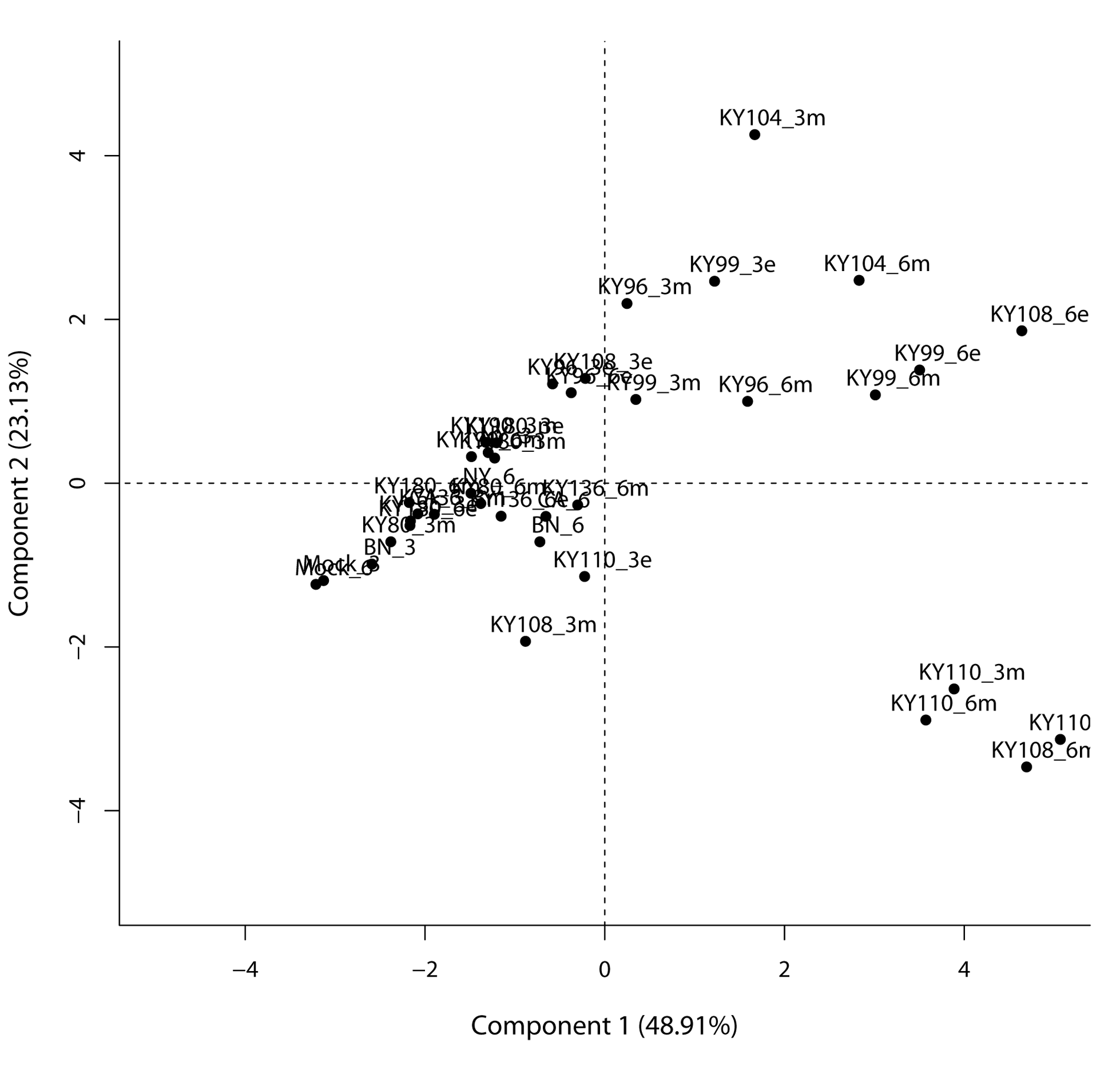

Supplement: Figure S1 — Principal Components Analysis (PCA) of mouse lung cytokine and chemokine expression after challenge with clinical Influenza A (H1N1) virus isolates from Kentucky, 2009. Standardized mean values for each cytokine/chemokine are plotted from Day 3 and Day 6 post-infection (n = 3, each) against the first two principal components, accounting for 72% of the variation in the analysis. (TIF) [file pone.0056602.s001.tif]

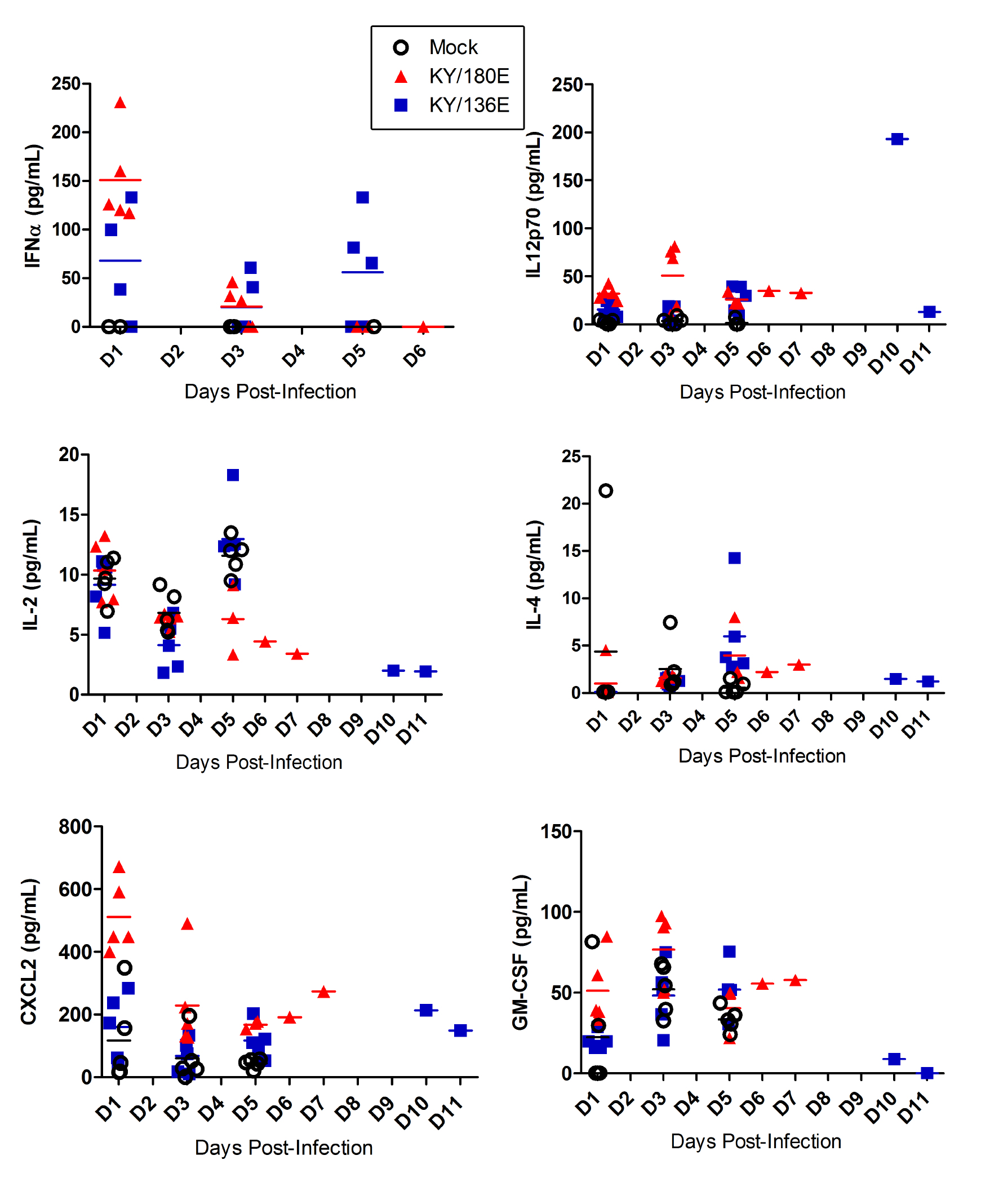

Supplement: Figure S2 — Cytokine and chemokine profiles from mouse lung homogenate. DBA/2 mice were infected with KY/180E, KY/136E (105 pfu), or mock-infected with PBS. The mice were sacrificed 1, 3, and 5 days post-infection (n = 5 mice per group-day). Samples from moribund mice taken after Day 5 post-infection were also analyzed when available. Bars indicate mean concentration. (TIF) [file pone.0056602.s002.tif]

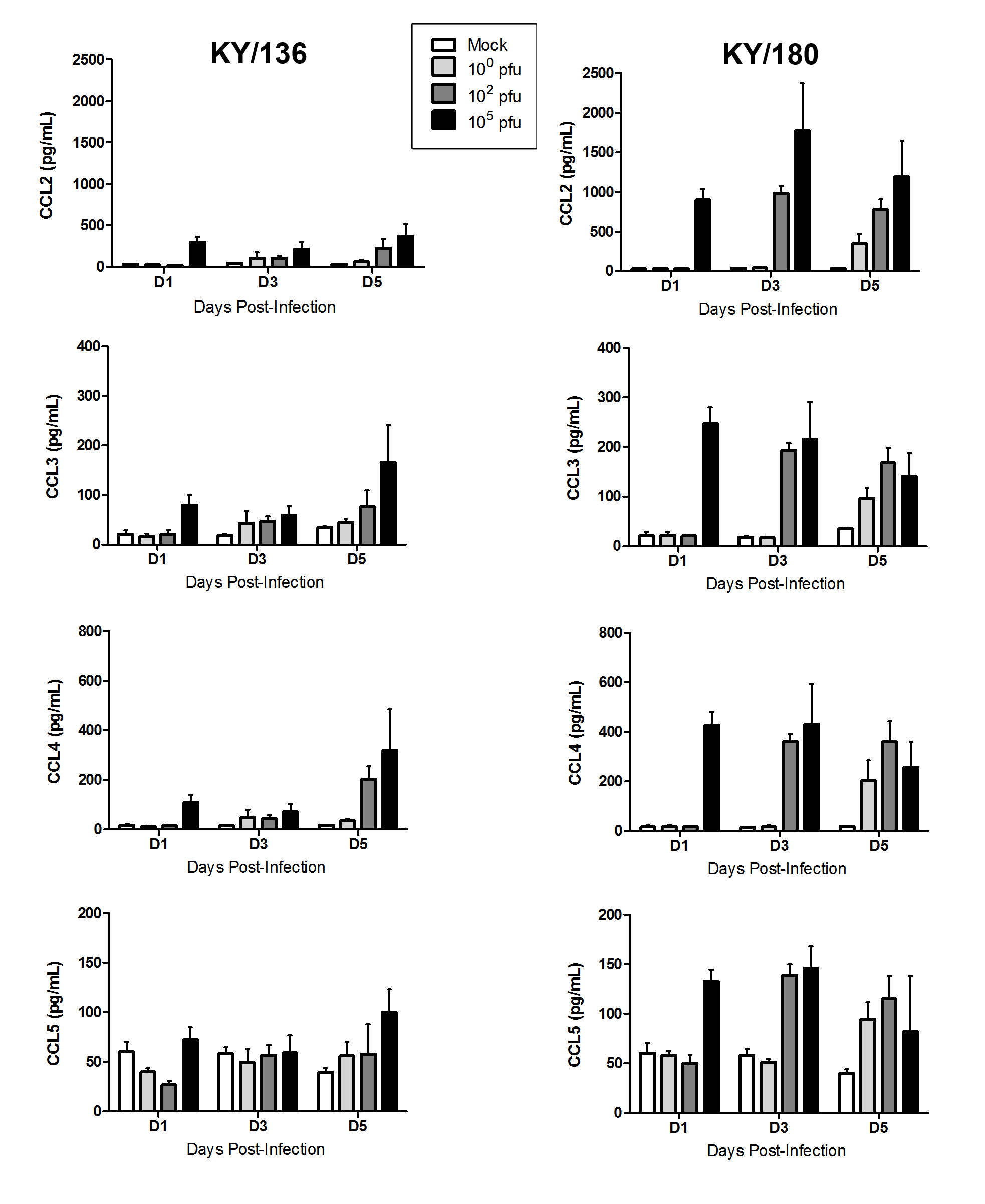

Supplement: Figure S3 — (TIF) [file pone.0056602.s003.tif]

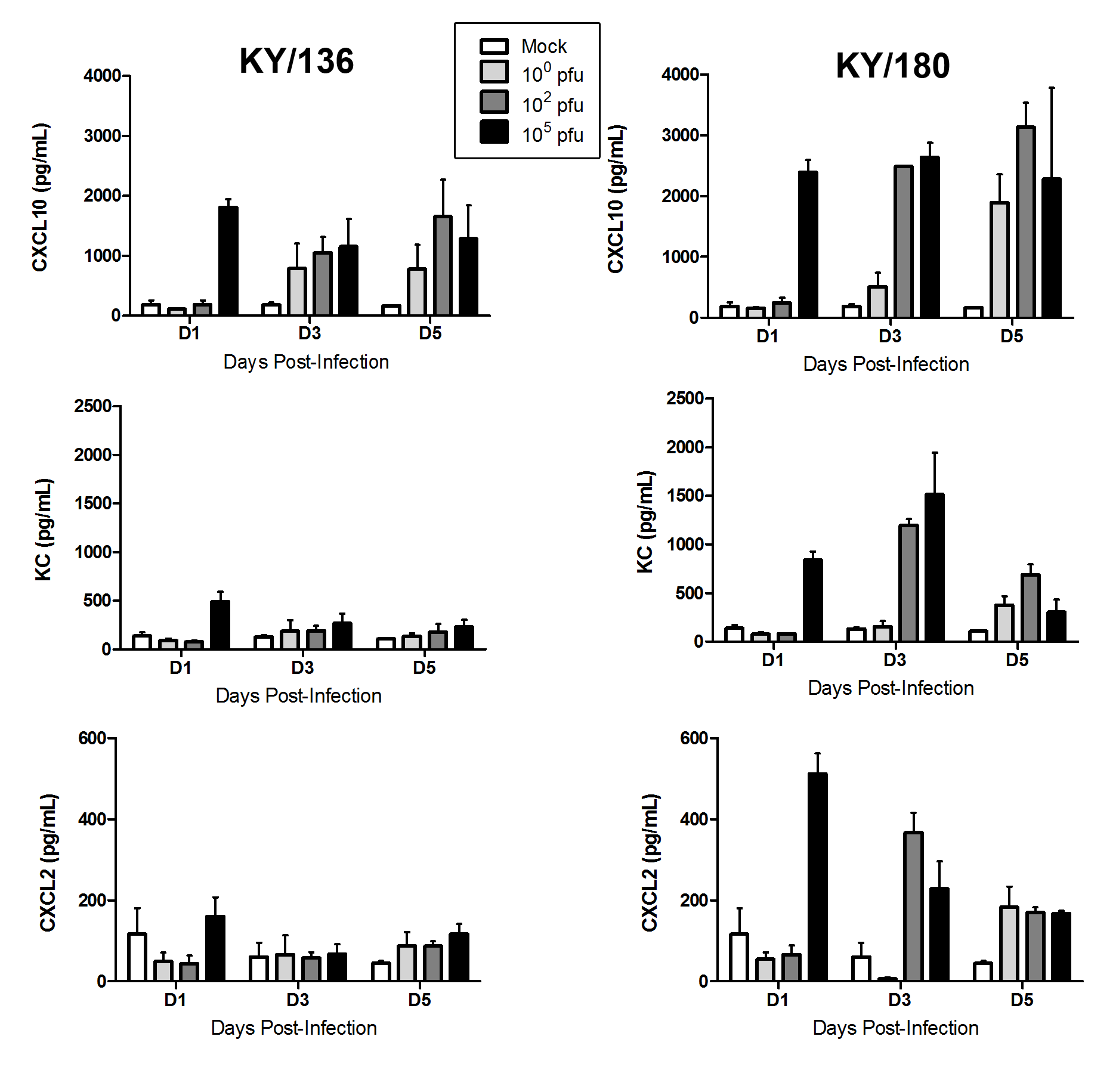

Supplement: Figure S4 — Dose response of chemokines in the lungs of mice infected with KY/136 or KY/180 influenza A (H1N1) virus isolates. Mice were infected with 100, 102, or 105 pfu of virus and samples were collected upon euthanasia on days 1, 3, or 5 post-challenge (D1, D3, and D5, respectively). n = 5 mice per dose-day for all groups except for mice infected with 105 pfu of KY/180 where only 2/5 mice survived to D5. (TIF) [file pone.0056602.s004.tif]

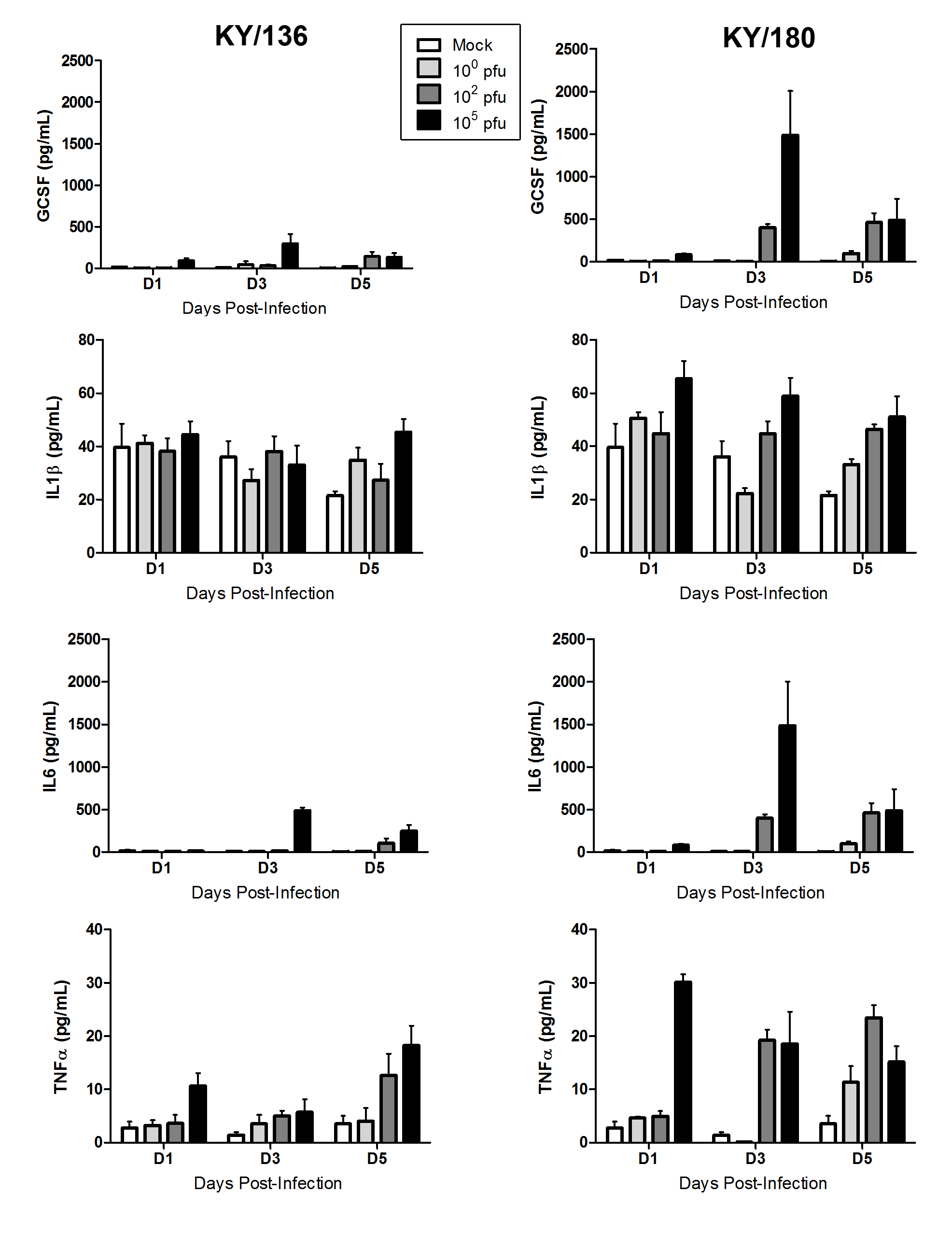

Supplement: Figure S5 — Dose response of cytokines in the lungs of mice infected with KY/136 or KY/180 influenza A (H1N1) virus isolates. Mice were infected with 100, 102, or 105 pfu of virus and samples were collected upon euthanasia on days 1, 3, or 5 post-challenge (D1, D3, and D5, respectively). n = 5 mice per dose-day for all groups except for mice infected with 105 pfu of KY/180 where only 2/5 mice survived to D5. (TIF) [file pone.0056602.s005.tif]

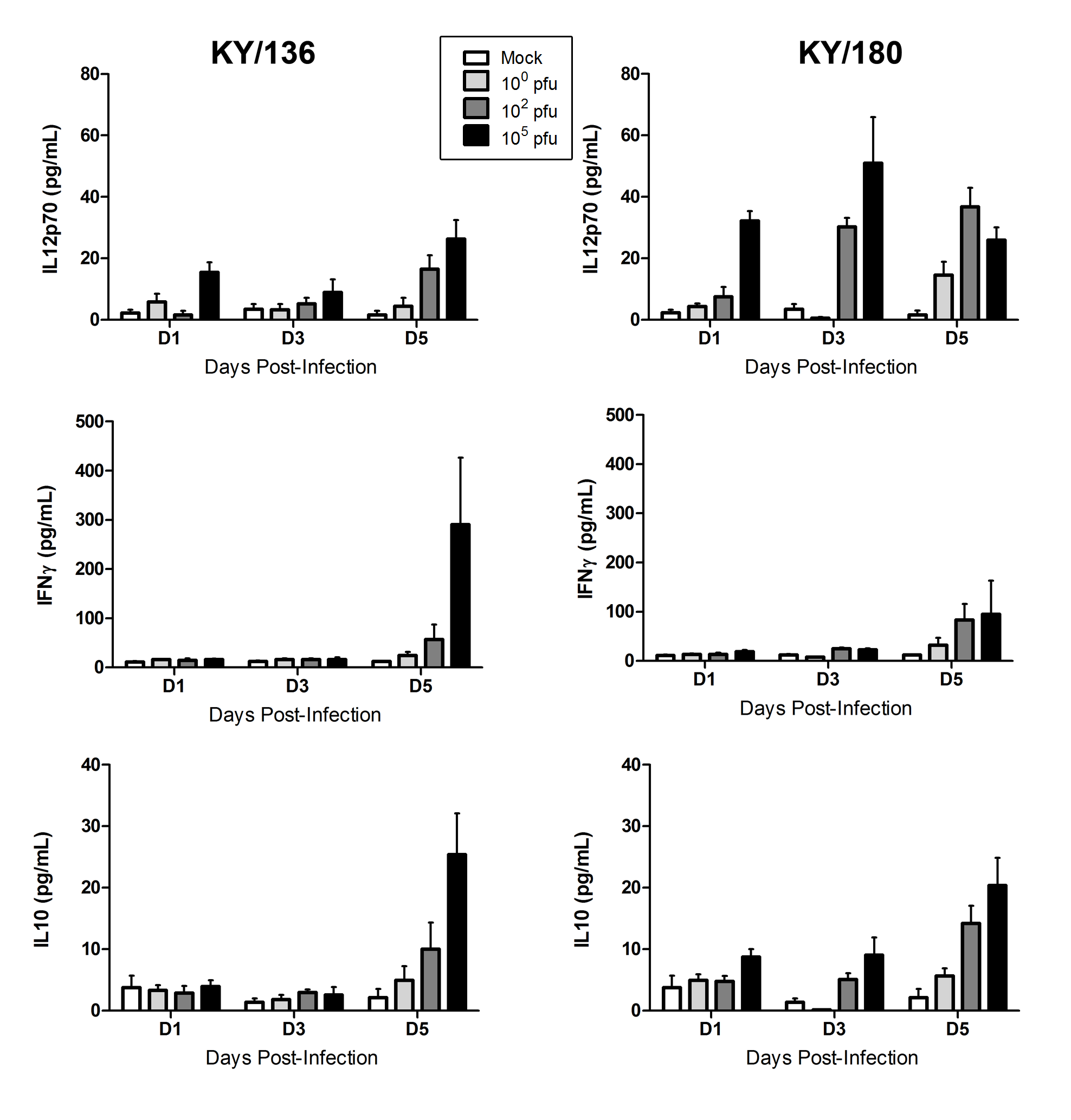

Supplement: Figure S6 — Dose response of cytokines in the lungs of mice infected with KY/136 or KY/180 influenza A (H1N1) virus isolates. Mice were infected with 100, 102, or 105 pfu of virus and samples were collected upon euthanasia on days 1, 3, or 5 post-challenge (D1, D3, and D5, respectively). n = 5 mice per dose-day for all groups except for mice infected with 105 pfu of KY/180 where only 2/5 mice survived to D5. (TIF) [file pone.0056602.s006.tif]

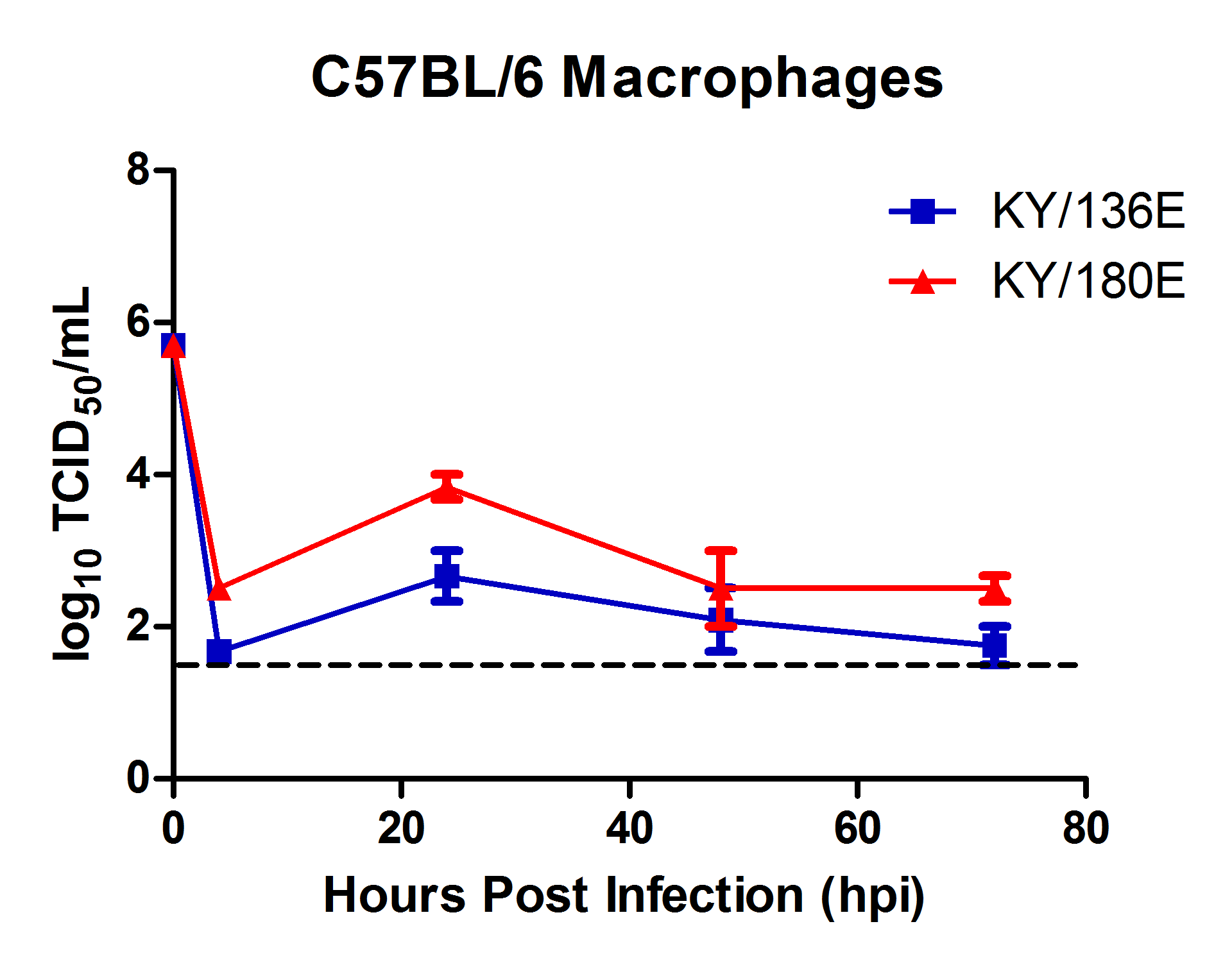

Supplement: Figure S7 — Replication kinetics of KY/180E and KY/180E isolates in C57BL/6 mouse macrophages. The macrophage cell line, BEIR #NR-9465, was infected at 1.0 MOI (two independent experiments at n = 3 per experiment) and clarified supernatants were taken at 4, 24, 48, and 72 hour post-infection. Virus titers were measured by TCID50 assay on MDCK cells. (TIF) [file pone.0056602.s007.tif]
