# Supplementary material for: Phenotypic Differences in Virulence and Immune Response in Closely Related Clinical Isolates of Influenza A 2009 H1N1 Pandemic Viruses in Mice
Source: PLoS One. 2013 Feb 18;8(2):e56602. doi: 10.1371/journal.pone.0056602 (PMC3575477; doi:10.1371/journal.pone.0056602)
Supplement: Table S1 — Comorbidities associated with severe, hospitalized influenza pneumonia patients. (DOCX) [file pone.0056602.s008.docx]

**Table S1.** Comorbidities associated with severe, hospitalized influenza pneumonia patients.

| **ID ID** | **Gender** | **BMI** | **Tamiflu**  **Therapy** | **PSI** | **PSI Class** | **COPD** | **Diab** | **Cancer** | **Other**  **Infect** | **Invasive**  **Vent Fail** |
| --- | --- | --- | --- | --- | --- | --- | --- | --- | --- | --- |
| 80 | Female | 123.6 | Tamiflu  75 mg Q12H x 7 days | 61 | II | yes | no | no | MRSA | yes |
| 96 | Male | 21.9 | No | 91 | IV | no | no | no | None | yes |
| 99 | Female | 76.90 | No-Flu vaccine only | 54 | II | no | no | no | None | 0 |
| 104 | Male | 26.63 | Tamiflu 75mg x2 doses | 123 | IV | no | yes | no | *Proteusspp.* | yes |
| 108 | Female | 79.71 | Tamiflu  75 mg daily x 5 days | 106 | IV | yes | yes | no | None | no |
| 110 | Female | 21.40 | Tamiflu  75 mg daily x 5 days | 73 | III | no | yes | no | None | no |
| 136 | Male | 24.4 | Tamiflu 150mg daily x 5 days | 76 | III | no | yes | yes | MRSA | yes |
| 180 | Male | 49.4 | Tamiflu | 93 | IV | yes | no | no | None | yes |
| 190 | Female | 56.4 | Tamiflu | 75 | III | yes | yes | no | none | no |
| Legend: BMI, body mass index; PSI, pneumonia severity index**;** COPD, Chronic obstructive pulmonary syndrome; MRSA, Methicillin-resistant *Staphylococcus aureus***;** Inf, infection; vent, ventilation failure | | | | | | | | | | |
