# Supplementary material for: Phenotypic Differences in Virulence and Immune Response in Closely Related Clinical Isolates of Influenza A 2009 H1N1 Pandemic Viruses in Mice
Source: PLoS One. 2013 Feb 18;8(2):e56602. doi: 10.1371/journal.pone.0056602 (PMC3575477; doi:10.1371/journal.pone.0056602)
Supplement: Table S2 — Variants noted in amino acid sequence alignments of H1N1pdm clinical isolates. (DOCX) [file pone.0056602.s009.docx]

**Table S2.** Variants noted in amino acid sequence alignments of H1N1pdm clinical isolates.

|  | **M1** | **HA1** | | | | | | | | | | | | | | | | | | | | | |  | | |  | | | | |  | | | |  |  |
| --- | --- | --- | --- | --- | --- | --- | --- | --- | --- | --- | --- | --- | --- | --- | --- | --- | --- | --- | --- | --- | --- | --- | --- | --- | --- | --- | --- | --- | --- | --- | --- | --- | --- | --- | --- | --- | --- |
| **A.A. Residue** | **30** | **39** | | **83** | | **83** | | **84** | | **97** | | **116** | | **121** | | **137** | | **147** | | **183** | | **186** | | **199** | | | **200** | | | **203** | | | |  |  |  |  |
| **A.A. Change** | **S🡪G** | **G🡪E** | | **S🡪P** | | **S🡪F** | | **S🡪N** | | **D🡪N** | | **I🡪M** | | **S🡪G** | | **P🡪T** | | **N🡪S** | | **S🡪P** | | **A🡪T** | | **V🡪A** | | | **F🡪L** | | | **T🡪S** | | | |  |  |  |  |
| CA/07 | A | G | | **C** | | C | | G | | G | | A | | **A** | | C | | A | | T | | G | | T | | | T | | | **T** | | | |  |  |  |  |
| NY/18 | A | G | | T | | C | | G | | G | | A | | A | | C | | A | | T | | G | | T | | | T | | | A | | | |  |  |  |  |
| NL/602 | A | G | | T | | C | | G | | G | | A | | A | | C | | A | | T | | G | | T | | | T | | | **T** | | | |  |  |  |  |
| KY/80 | A | G | | T | | C | | G | | G | | A | | **G** | | C | | A | | T | | G | | T | | | T | | | A | | | |  |  |  |  |
| KY/96 | A | G | | T | | C | | G | | G | | A | | A | | C | | A | | T | | G | | T | | | T | | | **T** | | | |  |  |  |  |
| KY/99 | A | G | | T | | C | | G | | G | | **G** | | A | | C | | A | | T | | G | | **C** | | | T | | | A | | | |  |  |  |  |
| KY/104 | A | G | | T | | C | | G | | **A** | | A | | A | | C | | A | | T | | G | | T | | | T | | | A | | | |  |  |  |  |
| KY/110 | A | **A** | | T | | C | | G | | G | | A | | A | | **A** | | A | | T | | G | | T | | | T | | | A | | | |  |  |  |  |
| KY/136/E | A | G | | T | | **T** | | G | | G | | A | | A | | C | | A | | T | | G | | T | | | T | | | A | | | |  |  |  |  |
| KY/180/E | **G** | G | | **C** | | C | | **A** | | G | | A | | A | | C | | A | | **C** | | **A** | | T | | | T | | | A | | | |  |  |  |  |
| KY/180/M | NA | G | | **C** | | C | | **A** | | G | | A | | A | | C | | **G** | | **C** | | **A** | | T | | | T | | | A | | | |  |  |  |  |
| KY/190 | NA | G | | **C** | | C | | **A** | | G | | A | | A | | C | | **G** | | T | | G | | T | | | **C** | | | A | | | |  |  |  |  |
|  | **HA1** | | | | | | | | | | **HA2** | | | | | | **PA** | | | | | | | | | | | | | | | | | | | |  |
| **A.A. Residue** | **222** | | **223** | | **234** | | **293** | | **321** | | **374** | | **440** | | **499** | | **14** | | **387** | | **465** | | **471** | | **538** | | | **581** | | | | | **610** | | | |  |
| **A.A. Change** | **D🡪G** | | **Q🡪R** | | **V🡪I** | | **Q🡪H** | | **V🡪I** | | **E🡪K** | | **S🡪L** | | **E🡪K** | | **V🡪I** | | **V🡪I** | | **I🡪T** | | **N🡪S** | | **E🡪K** | | | **M🡪L** | | | | | **E🡪D** | | | |  |
| CA/07 | A | | A | | G | | G | | **A** | | G | | C | | G | | G | | G | | T | | A | | G | | | **C** | | | | | A | | | |  |
| NY/18 | A | | **R** | | G | | G | | G | | G | | C | | G | | G | | G | | T | | A | | G | | | A | | | | | A | | | |  |
| NL/602 | A | | A | | G | | G | | G | | G | | C | | G | | G | | G | | T | | A | | G | | | **C** | | | | | A | | | |  |
| KY/80 | A | | A | | G | | G | | **A** | | G | | C | | G | | G | | G | | T | | A | | G | | | A | | | | | A | | | |  |
| KY/96 | A | | A | | G | | **T** | | G | | G | | C | | **A** | | G | | **A** | | T | | A | | G | | | A | | | | | A | | | |  |
| KY/99 | A | | A | | G | | G | | G | | G | | C | | G | | G | | G | | T | | **G** | | G | | | A | | | | | A | | | |  |
| KY/104 | A | | A | | G | | G | | G | | **A** | | C | | G | | G | | G | | T | | A | | **A** | | | A | | | | | A | | | |  |
| KY/110 | A | | A | | G | | G | | G | | G | | C | | G | | NA | | NA | | NA | | NA | | NA | | | A | | | | | NA | | | |  |
| KY/136/E | A | | A | | **A** | | G | | G | | G | | C | | G | | G | | G | | **C** | | A | | G | | | A | | | | | A | | | |  |
| KY/180/E | **G** | | A | | G | | G | | G | | **A** | | C | | G | | **A** | | G | | T | | A | | G | | | A | | | | | **T** | | | |  |
| KY/180/M | **G** | | A | | G | | G | | G | | **A** | | C | | G | | NA | | NA | | NA | | NA | | NA | | | NA | | | | | NA | | | |  |
| KY/190 | A | | A | | G | | G | | G | | **A** | | **T** | | G | | NA | | NA | | NA | | NA | | NA | | | NA | | | | | NA | | | |  |
|  | **PA** | | | | | |  | | **PA-X** | |  | | **PB1** | | | | | | | | **PB2** | | | | | | | | | | | | | | | |  |
| **A.A. Residue** | **647** | | **654** | | **716** | | **14** | | **196** | | **215** | | **83** | | **563** | | **662** | | **736** | | **33** | | **176** | | **183** | | | **340** | | | | | **584** | | | |  |
| **A.A. Change** | **N🡪D** | | **Q🡪E** | | **K🡪Q** | | **V🡪I** | | **E🡪G** | | **P🡪Q** | | **A🡪G** | | **R🡪K** | | **T🡪N** | | **K🡪G** | | **K🡪R** | | **I🡪T** | | **L🡪M** | | | **K🡪N** | | | | | **V🡪I** | | | |  |
| CA/07 | A | | C | | A | | G | | **G** | | C | | C | | G | | C | | A | | A | | T | | C | | | A | | | | | G | | | |  |
| NY/18 | A | | C | | A | | G | | A | | C | | C | | G | | C | | A | | A | | T | | C | | | A | | | | | G | | | |  |
| NL/602 | A | | C | | A | | G | | A | | C | | C | | G | | C | | A | | A | | T | | C | | | A | | | | | G | | | |  |
| KY/80 | A | | C | | A | | G | | A | | C | | C | | G | | C | | A | | A | | T | | C | | | A | | | | | G | | | |  |
| KY/96 | A | | C | | A | | G | | A | | **A** | | C | | G | | C | | A | | **G** | | T | | **A** | | | A | | | | | G | | | |  |
| KY/99 | **G** | | **S** | | A | | G | | A | | C | | C | | G | | C | | A | | A | | T | | C | | | A | | | | | G | | | |  |
| KY/104 | A | | C | | A | | G | | A | | C | | C | | **A** | | C | | A | | A | | T | | C | | | A | | | | | G | | | |  |
| KY/110 | NA | | NA | | NA | | G | | A | | C | | C | | G | | **A** | | A | | A | | **C** | | C | | | A | | | | | G | | | |  |
| KY/136/E | A | | C | | A | | G | | A | | C | | **G** | | G | | C | | A | | A | | T | | C | | | **T** | | | | | G | | | |  |
| KY/180/E | A | | C | | **C** | | **A** | | A | | C | | C | | G | | C | | **G** | | A | | T | | C | | | A | | | | | **A** | | | |  |
|  | **NA** | | | | | | | | | | | | | | | **NP** | | | | | | **NS1** | | | | | | |  | | | | | |  |  |  |
| **A.A. Residue** | **79** | **106** | | **108** | | **150** | | **220** | | **248** | | **396** | | **407** | | **100** | | **181** | | **373** | | **112** | | **123** | | **154** | | | | |  |  |  |  |  |  |  |
| **A.A. Change** | **S🡪P** | **I🡪V** | | **I🡪V** | | **K🡪R** | | **R🡪K** | | **D🡪N** | | **I🡪K** | | **V🡪I** | | **I🡪V** | | **A🡪D** | | **T🡪I** | | **I🡪M** | | **V🡪I** | | **G🡪R** | | | | |  |  |  |  |  |  |  |
| CA/07 | T | **G** | | A | | A | | G | | **A** | | T | | G | | **G** | | C | | C | | A | | G | | G | | | | |  |  |  |  |  |  |  |
| NY/18 | T | A | | A | | A | | G | | G | | T | | G | | A | | C | | C | | A | | G | | G | | | | |  |  |  |  |  |  |  |
| NL/602 | T | A | | **G** | | A | | G | | **A** | | T | | **A** | | **G** | | C | | **T** | | A | | G | | G | | | | |  |  |  |  |  |  |  |
| KY/80 | T | A | | A | | A | | G | | G | | T | | G | | A | | C | | C | | A | | G | | G | | | | |  |  |  |  |  |  |  |
| KY/96 | **C** | A | | A | | A | | G | | G | | **A** | | G | | A | | C | | C | | A | | **A** | | G | | | | |  |  |  |  |  |  |  |
| KY/99 | T | A | | A | | A | | G | | G | | T | | G | | A | | C | | C | | A | | G | | G | | | | |  |  |  |  |  |  |  |
| KY/104 | T | A | | A | | A | | G | | G | | T | | G | | A | | C | | C | | A | | G | | G | | | | |  |  |  |  |  |  |  |
| KY/110 | T | A | | A | | A | | G | | G | | T | | G | | A | | C | | C | | A | | G | | **A** | | | | |  |  |  |  |  |  |  |
| KY/136/E | T | A | | A | | **G** | | G | | G | | T | | G | | A | | **A** | | C | | **G** | | G | | G | | | | |  |  |  |  |  |  |  |
| KY/180/E | T | A | | A | | A | | **A** | | G | | T | | G | | A | | C | | C | | A | | G | | G | | | | |  |  |  |  |  |  |  |

*NS = not yet sequenced; for KY/190 only the HA gene has been sequenced.
