# Supplementary material for: Phenotypic Differences in Virulence and Immune Response in Closely Related Clinical Isolates of Influenza A 2009 H1N1 Pandemic Viruses in Mice
Source: PLoS One. 2013 Feb 18;8(2):e56602. doi: 10.1371/journal.pone.0056602 (PMC3575477; doi:10.1371/journal.pone.0056602)
Supplement: Table S3 — GenBank accession numbers of H1N1pdm isolates. (DOCX) [file pone.0056602.s010.docx]

**Table S3.** GenBank accession numbers of H1N1pdm isolates.

|  | **HA** | **M** | **NA** | **NP** | **NS** | **PA** | **PB1** | **PB2** |
| --- | --- | --- | --- | --- | --- | --- | --- | --- |
| **A/California/07/09** | FJ969540 | FJ966975 | FJ984386 | FJ969536 | FJ969528 | FJ966977 | FJ969531 | FJ984387 |
| **A/NewYork/18/09** | GQ232064 | FJ984348 | FJ984350 | FJ984352 | GQ232063 | FJ984354 | FJ984353 | FJ984351 |
| **A/Netherlands/602/09** | CY039527 | CY046944 | CY039528 | CY046943 | CY046945 | CY046942 | CY046941 | CY046940 |
| **A/Kentucky/80/09/E** | JX875011 | JX875005 | JX875008 | JX875012 | JX875010 | JX875009 | JX875006 | JX875007 |
| **A/Kentucky/96/09/E** | JX875039 | JX875033 | JX875036 | JX875040 | JX875038 | JX875037 | JX875034 | JX875035 |
| **A/Kentucky/99/09/E** | JX875019 | JX875014 | JX875016 | JX875020 | JX875018 | JX875017 | JX875015 | * |
| **A/Kentucky/104/09/E** | JX875047 | JX875041 | JX875044 | JX875048 | JX875046 | JX875045 | JX875042 | JX875043 |
| **A/Kentucky/110/09/E** | JX875054 | JX875049 | JX875052 | JX875055 | JX875053 | * | JX875050 | JX875051 |
| **A/Kentucky/136/10/E** | CY099330 | JX875021 | CY099331 | JX875026 | JX875025 | JX875024 | JX875022 | JX875023 |
| **A/Kentucky/180/10/M** | JX875056 | * | * | * | * | * | * | * |
| **A/Kentucky/180/10/E** | CY099332 | JX875027 | CY099333 | JX875032 | JX875031 | JX875030 | JX875028 | JX875029 |
| **A/Kentucky/190/10/E** | JX875013 | * | * | * | * | * | * | * |

* not yet sequenced
