# Supplementary material for: Phenotypic Differences in Virulence and Immune Response in Closely Related Clinical Isolates of Influenza A 2009 H1N1 Pandemic Viruses in Mice
Source: PLoS One. 2013 Feb 18;8(2):e56602. doi: 10.1371/journal.pone.0056602 (PMC3575477; doi:10.1371/journal.pone.0056602)
Supplement: Table S4 — Virus titers (TCID50/ml*) on 1, 3, and 5 days post-infection in lungs and nasal turbinates of DBA/2 mice infected with KY/180E and KY/136E. (DOCX) [file pone.0056602.s011.docx]

|  |  | **Lung** | | | **NT** | | |
| --- | --- | --- | --- | --- | --- | --- | --- |
|  | **Dose** | **1 DPI** | **3 DPI** | **5 DPI** | **1 DPI** | **3 DPI** | **5 DPI** |
| KY/136E | 10^0^ | < 1.5* | 2.86 ± 2.15 | 0.68 ± 1.52 | < 1.5 | < 1.5 | < 1.5 |
|  | 10^2^ | 1.42 ± 1.95 | 3.78 ± 2.23 | 2.50 ± 0.00 | < 1.5 | < 1.5 | 1.94 ± 1.89 |
|  | 10^5^ | 5.04 ± 0.86 | 3.64 ± 2.17 | < 1.5 | < 1.5 | 3.64 ± 0.76 | 2.92 ± 0.29 |
| KY/180E | 10^0^ | 0.50 ± 1.12 | 2.76 ± 2.61 | 4.40 ± 2.47 | < 1.5 | < 1.5 | < 1.5 |
|  | 10^2^ | 4.86 ±1.77 | 6.54 ± 0.36 | 6.42 ± 0.44 | 0.50 ± 1.12 | 5.22± 0.54 | 5.32 ± 0.75 |
|  | 10^5^ | 7.54 ± 0.34 | 6.18 ± 0.46 | 6.38 ± 0.30 | 5.40 ± 0.26 | 4.80 ± 0.45 | 4.96 ± 0.36 |

Legend: Dose = pfu/mouse. Values in mean log_10_ TCID_50_/mL ± S.D. (*n* = 5 mice per dose-day);

* Limit of detection = 10^1.5^ TCID_50_ / mL.
