# Supplementary material for: Phenotypic Differences in Virulence and Immune Response in Closely Related Clinical Isolates of Influenza A 2009 H1N1 Pandemic Viruses in Mice
Source: PLoS One. 2013 Feb 18;8(2):e56602. doi: 10.1371/journal.pone.0056602 (PMC3575477; doi:10.1371/journal.pone.0056602)
Supplement: Table S5 — Relative magnitude of immune responses of H1N1pdm isolates in mice in groups revealed by principal component analysis clustering. (DOCX) [file pone.0056602.s012.docx]

| **Cytokine/Chemokine** | **Function** | **DPI** | **Group 1** | **Group 2** | **Group 3** | **Group 4** |
| --- | --- | --- | --- | --- | --- | --- |
| IL-6 | Inflammation | 3 | + | +++ | + | + |
|  |  | 6 | + | ++++ | +++ | + |
| TNFα | Inflammation | 3 | + | +++ | +++ | ++ |
|  |  | 6 | + | +++ | ++++ | + |
| KC | PMN attraction | 3 | + | +++ | ++ | ++ |
|  |  | 6 | + | +++ | ++ | + |
| G-CSF | PMN proliferation | 3 | + | +++ | ++ | +++ |
|  |  | 6 | + | ++ | ++ | ++ |
| CXCL9 (MIG) | T-cell chemoattractant | 3 | +++ | ++++ | ++ | +++ |
|  |  | 6 | ++++ | +++ | ++ | + |
| CCL2 (MCP-1) | Monocyte chemoattractant | 3 | + | +++ | ++++ | ++ |
|  |  | 6 | ++ | +++ | ++++ | ++ |
| IL-1β | Inflammation | 3 | + | ++ | ++ | + |
|  |  | 6 | ++ | ++ | +++ | + |
| CXCL3 (MIP1α) | PMN attraction | 3 | + | ++ | +++ | + |
|  |  | 6 | ++ | +++ | ++++ | + |
| CXCL10 (IP10) | Chemoattractant | 3 | ++ | - | ++++ | ++ |
|  |  | 6 | +++ | + | ++++ | + |
| IFNγ | Adaptive immunity | 3 | - | - | + | - |
|  |  | 6 | + | ++ | +++ | - |
| IL-10 | Anti-inflammation | 3 | - | - | + | - |
|  |  | 6 | + | ++ | +++ | - |

Legend: Group 1, BN/59, CA/07, KY/80, KY/136; Group 2, KY/96, KY/99, KY/104, KY/108; Group 3, KY/108, KY/110; Group 4, KY/180, KY/190.
