# Supplementary material for: Phenotypic Differences in Virulence and Immune Response in Closely Related Clinical Isolates of Influenza A 2009 H1N1 Pandemic Viruses in Mice
Source: PLoS One. 2013 Feb 18;8(2):e56602. doi: 10.1371/journal.pone.0056602 (PMC3575477; doi:10.1371/journal.pone.0056602)
Supplement: Table S6 — Summary of references to mutations in Influenza A (H1N1) isolates with observed virulence. (DOCX) [file pone.0056602.s013.docx]

| **Gene product** | **Mutation** | **Source** | **Citation** |
| --- | --- | --- | --- |
| HA1 | S83P | mouse*, in vitro* | 3, 12 |
| HA1 | S183P | mouse | 21, 22, 93, 94 |
| HA1 | T203S | mouse | 44 |
| HA1 | D222G | human, mouse, macaque | 10, 30, 31, 32, 34, 36, 38, 37, 43, 89 |
| HA1 | Q293H | mouse | 44 |
| HA1 | V321I | mouse | 3, 24, 44 |
| HA2 | E374K | human | 95 |
| NA | I106A | mouse | 44 |
| NS1 | V123I | mouse | 44 |
| PA | V14I | mouse | 44 |
| PB2 | K340N | human | 95 |
